# Repeatedly occurring retrograde menstruation intensifies central sensitization driven by neuroinflammation in endometriosis models

**DOI:** 10.1172/JCI194136

**Published:** 2026-03-17

**Authors:** Madeleine E. Harvey, Mingxin Shi, Yeongseok Oh, Taylor M. Page, Debra A. Mitchell, Addie Luo, Ov D. Slayden, James A. MacLean, Anjali Sharma, Kanako Hayashi

**Affiliations:** 1 School of Molecular Biosciences, Center for Reproductive Biology, Washington State University, Pullman, Washington, USA.; 2 Division of Reproductive and Developmental Sciences, Oregon National Primate Research Center, Oregon Health & Science University, Beaverton, Oregon, USA.; 3 Department of Chemistry, Washington State University, Pullman, Washington, USA.

**Keywords:** Inflammation, Reproductive biology, Macrophages

## Abstract

This study investigated how chronic pelvic pain (CPP) develops using rhesus macaques with naturally occurring endometriosis and a multiple lesion induction mouse model (MIM), as repeated retrograde menstruation is considered an underlying mechanism of endometriosis pathogenesis. MIM increased lesion numbers and elevated hypersensitivity. Elevated persistent glial cell activation was observed across multiple brain regions or spinal cords in MIM and rhesus macaques. Elevated TRPV1, SP, and CGRP expressions in the dorsal root ganglia (DRG) were persistent in MIM. MIM induced the severe disappearance of TIM4^hi^MHCII^lo^ residential macrophages and an influx of increased pro-inflammatory TIM4^lo^MHCII^hi^ macrophages in the peritoneal cavity. Cytokine levels were persistently elevated in MIM. Furthermore, dienogest (a synthetic progestin) and fingolimod (a selective immunosuppressor) reduced hyperalgesia and neuroinflammation. Our results indicate that recurrent retrograde menstruation can be a peripheral stimulus that induces nociceptive pain and creates a composite chronic inflammatory stimulus, leading to neuroinflammation and sensitization of the central nervous system. The circuits of neuroplasticity and stimulation of peripheral organs via a feedback loop of neuroinflammation may mediate widespread endometriosis-associated CPP. These findings in mice were further supported by results from the spontaneously developed advanced endometriosis in rhesus macaques via recurrent retrograde menstruation.

## Abstract 2

Repeatedly occurring retrograde menstruation intensifies central sensitization driven by neuroinflammation in rhesus macaques and a mouse model of endometriosis.

## Graphical abstract

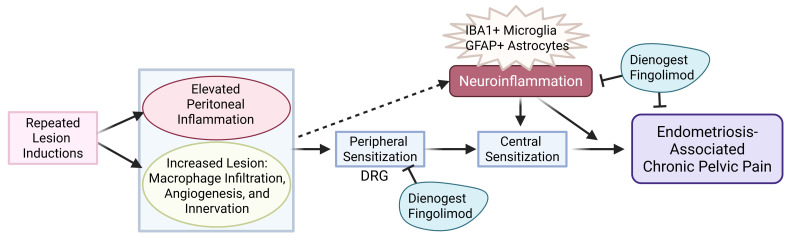

## Introduction

Endometriosis is a chronic inflammatory disease characterized by the presence of endometrium-like tissues outside the uterus (1) that affects approximately 10% of reproductive-aged women, representing approximately 190 million women worldwide (2, 3). It can cause debilitating chronic pelvic pain (CPP), manifesting as dysmenorrhea, dyschezia, dysuria, dyspareunia, and acyclic pelvic pain that dramatically reduces the quality of life of women (4–7). Many women can endure symptoms for several decades due to the onset of endometriosis-associated pain during adolescence (3) and have a greater risk of chronic opioid use for pain relief (8). Despite a sizeable clinical burden, the pathogenesis of endometriosis is complicated and remains poorly understood. The current medical treatment/management is noncurative. It is limited to surgical excision of endometriotic lesions or hormonal treatments to suppress estrogen production and action due to endometriosis being an estrogen-dependent disease. Surgical excision of lesions can alleviate endometriosis-associated pain, though pelvic pain frequently returns within a year of lesion removal, even in the absence of lesion regeneration (9, 10). Thus, endometriosis-associated CPP is not solely dependent on the presence of lesions (11).

Pain relies on peripheral stimuli to the spinal cord for processing and perception by the brain. Inflammatory mediators, such as pro-inflammatory cytokines and chemokines, prostaglandins, or nerve growth factor (NGF), evoke pain by directly activating and sensitizing nociceptor neurons in peripheral tissues and by modulating various ion channels, such as transient receptor potential ankyrin 1 (TRPA1), transient receptor potential vanilloid 1 (TRPV1), and voltage-gated sodium channels (12). Sensitized and activated nociceptors, specifically C-fibers, secrete neuropeptides like substance P (SP) and calcitonin gene-related peptide (CGRP) (13), which can trigger a positive feedback loop to stimulate pro-inflammatory mediator secretion, further perpetuating pain signaling (11). Through these processes of sensory signal transduction, increased neurotransmitter release, such as SP and CGRP, induces hyperactivity and hypersensitivity in the spinal cord and brain, known as central sensitization (14). In endometriosis, abundant immune responses are present at lesion (peripheral) sites, with increased levels of pro-inflammatory cytokines and chemokines throughout the pelvic cavity (15–18). Elevated tumor necrosis factor–α (TNF-α), interleukin-1β (IL-1β), and IL-6 levels have been reported in the peritoneal fluids and/or eutopic and ectopic endometrial tissues of women with endometriosis (17, 19–21). Specifically, TNF-α, IL-1β, CCL5, and NGF are elevated in the pelvic cavity of patients with endometriosis who reported CPP (22, 23). We have shown that TNF-α, IL-1β, and IL-6 are elevated in mouse peritoneal fluid following a single lesion induction (24, 25). Lesion induction increases SP, CGRP, and TRPV1 expression in the dorsal root ganglia (DRG) and elevates mechanical hyperalgesia and allodynia (24, 25). Thus, elevated inflammatory mediators sensitize nociceptor neurons in endometriotic lesions or pelvic organs; initiate pain stimuli; transmit them to the spinal cord and brain, thereby sensitizing the central nervous system (CNS); and induce endometriosis-associated pain. Although peripheral inflammation and sensitization explain some aspects of CPP, CPP can persist or recur in patients after lesion removal (26). Furthermore, the severity of pain is not correlated with lesion size, location, or extent of lesion infiltration into tissues (27). Chronic hyperexcitability perhaps induces long-lasting neuroplastic modification in the CNS.

Neuroinflammation is defined as an inflammatory response within the brain and spinal cord characterized by the infiltration of leukocytes, activation of glial cells, and production of pro-inflammatory cytokines and chemokines (12). Microglia and astrocytes are key regulators of inflammatory responses within the CNS, and their activation is not only a significant cause of neurologic and neurodegenerative diseases but also a painful insult (12, 28). CPP can also result from CNS top-down activation via neuroinflammation triggered by the dorsal root reflex in the spinal cord, which induces peripheral sensitization (12, 29). Retrograde menstruation, the reflux of menstrual tissues via the fallopian tube into the pelvic cavity, has been widely accepted as the origin of endometriotic lesions (30), and it causes massive inflammatory responses in the peritoneum. However, retrograde menstrual debris is cleared from the pelvic cavity by an innate immune response in the majority of women who do not develop endometriosis (11, 31). Yet menstrual cycles occur repeatedly in women. Each episode of retrograde menstruation induces a composite inflammatory response in the pelvic cavity, and unresolved inflammation is expected to worsen and develop into chronic conditions (11, 25). Thus, multiple chronic inflammatory stimuli are expected to enhance central sensitization and induce neuroinflammation in patients with endometriosis, thereby contributing to endometriosis-associated CPP.

In the present study, we carried out repeated cycles of lesion induction to examine whether multiple rounds of lesion induction mimic repeated retrograde menstruation and sensitize the CNS and whether they can drive neuroinflammation in a mouse model of endometriosis. We also examined mechanical hyperalgesia, peripheral inflammatory mediators and immune cells in the lesions and peritoneal fluid, and neurotransmitters in the DRG to understand how peripheral stimuli are associated with central sensitization and endometriosis-associated pain behavior. Furthermore, we examined markers of neuroinflammation in rhesus macaques with naturally occurring chronic endometriosis, as endometriosis is also associated with CPP in macaques (32). Macaques are nonhuman primates that menstruate, and the development and progression of spontaneous endometriosis are likely due to repeated retrograde menstrual cycles. Additionally, we investigated whether dienogest, a synthetic progestin, and fingolimod, an FDA-approved sphingosine 1-phosphate receptor-1 (S1PR1) modulator, can improve endometriosis-associated hyperalgesia and its mechanisms.

## Results

### Endometriosis lesion development by repeated cycles of lesion induction in mice.

**Figure 1:**
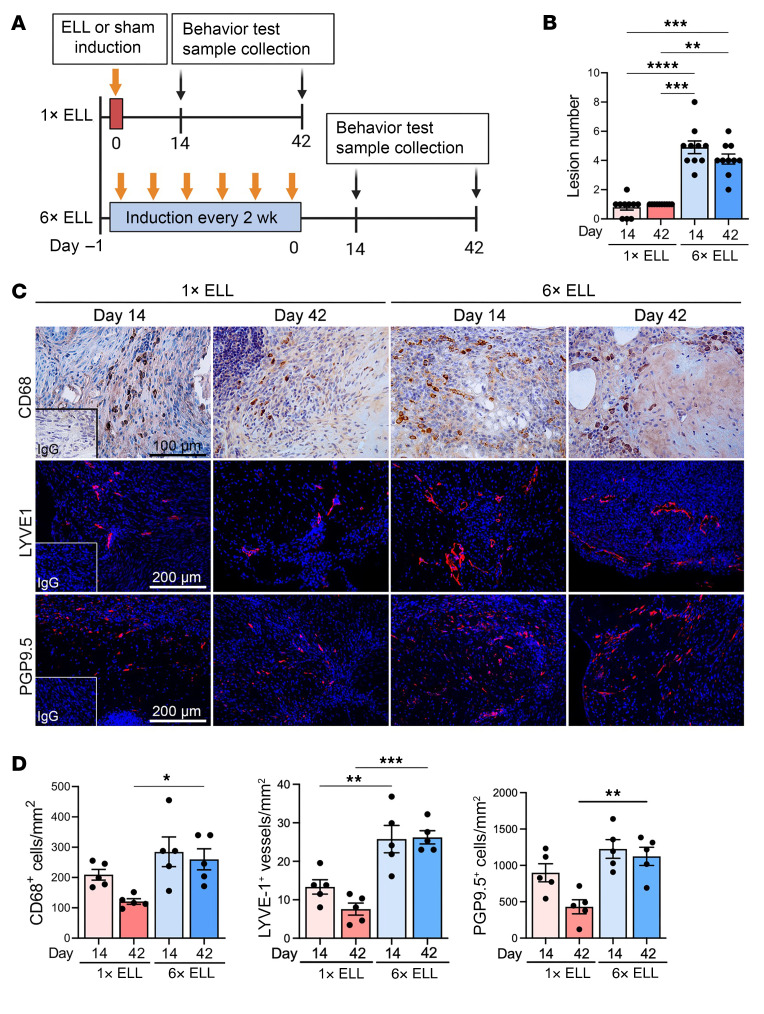
Multiple lesion induction mouse model of endometriosis. (**A**) Experimental design for study 1 as described in Methods. (**B**) Quantification of lesion numbers in single- or multiple-induction mice at 2 or 6 weeks after the last lesion induction (*n* = 10; 3 animals with no apparent lesions at day 14 in the single-induction group were included for behavioral assessment, shown in Figure 3). Representative immunohistochemical images (**C**) and quantification (**D**) of CD68^+^, LYVE1^+^, or PGP9.5^+^ cells in the lesions (*n* = 5). Following the Shapiro-Wilk normality test, the Kruskal-Wallis test was used to assess group differences in lesion numbers, whereas 1-way ANOVA followed by Tukey’s multiple-comparison test was used to analyze the numbers of CD68^+^, LYVE1^+^, or PGP9.5^+^ cells in the lesions among groups. Data are shown as the mean ± SEM. *P < 0.05, **P < 0.01, ***P < 0.001, ****P < 0.0001. ELL, endometriosis-like lesions.

We first assessed the effects of multiple inoculations of endometrial tissue on endometriotic lesion development and progression in a mouse model of endometriosis (Figure 1A). The experimental details of study 1 are described in the Methods. Lesion numbers were significantly increased in the multiple-induction mice at 2 weeks after the last lesion induction than in mice that received only a single induction (Figure 1B). These numbers remained higher in the multiple-induction mice at 6 weeks after lesion induction (Figure 1B). As macrophage infiltration is critical for lesion development, angiogenesis, and innervation (24, 25, 33), we next examined cell populations using established markers: macrophages (CD68), lymphatic endothelial cells (lymphatic vessel endothelial hyaluronan receptor 1, LYVE1), and nerve cells (protein gene product 9.5, PGP9.5) in the lesions (Figure 1, C and D). CD68^+^ macrophages were comparable in the single- and multiple-induction mice at 2 weeks, whereas more CD68^+^ macrophages were detected in the lesions with multiple inductions at 6 weeks (Figure 1, C and D). Significantly abundant LYVE1^+^ cells were observed in the multiple-induction mice compared with the single-induction mice at 2 and 6 weeks (Figure 1, C and D). Multiple-induction mice showed a more significant increase in PGP9.5^+^ nerve cells in the lesions than single-induction mice at 6 weeks, though there was no significant difference between the single- and multiple-induction mice at 2 weeks (Figure 1, C and D). These results suggest that multiple inductions enhance endometriotic lesion development and progression by increasing macrophage infiltration, angiogenesis/lymphangiogenesis, and innervation compared with a single induction. Macrophage infiltration and innervation remained greater in the multiple-induction mice for extended periods.

### Characterization of cellular components in rhesus macaques with spontaneous endometriosis.

**Figure 2:**
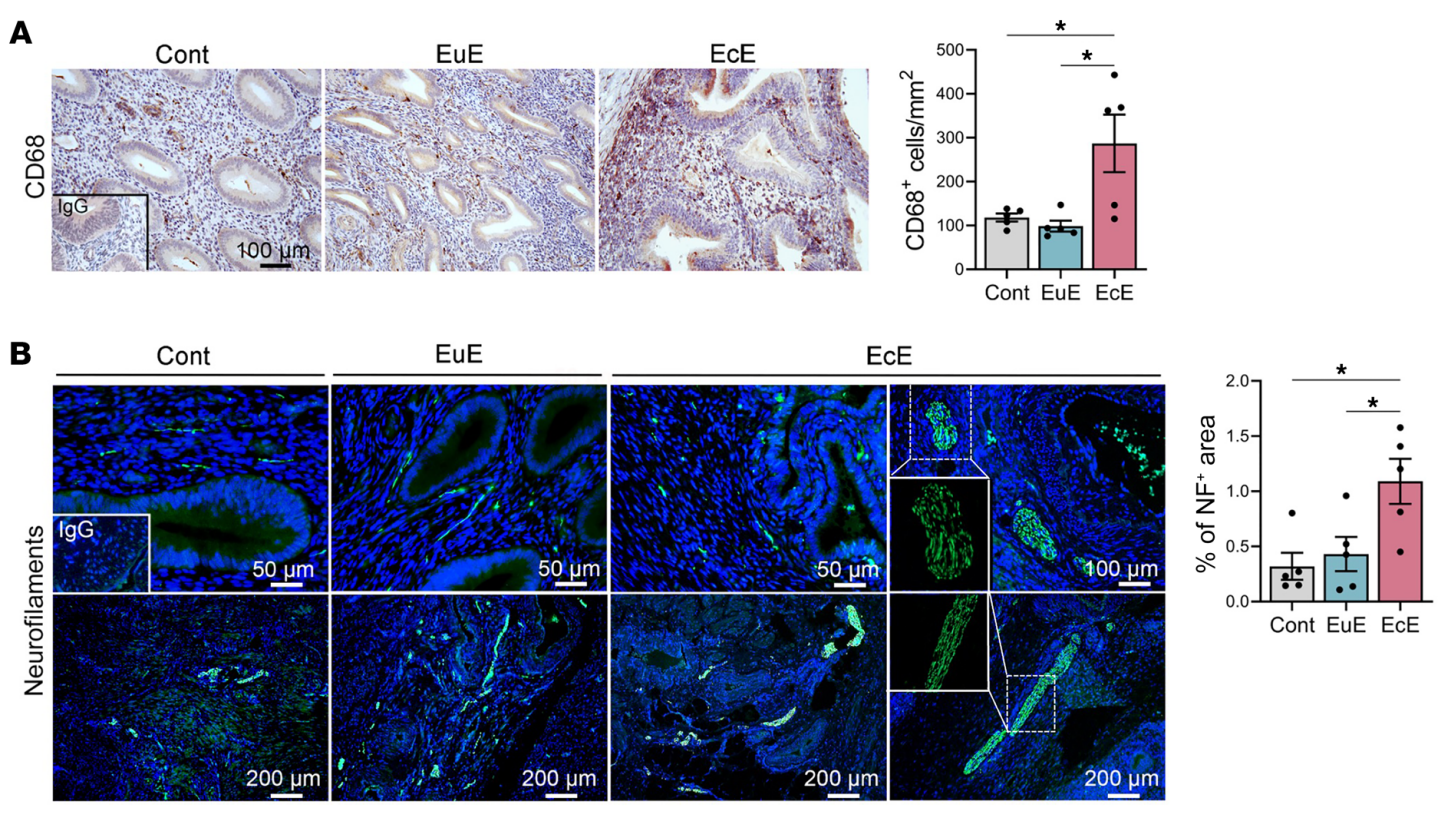
Spontaneously developed endometriosis in rhesus macaques. (**A**) Representative immunohistochemical images and quantification of CD68^+^ macrophages in the endometrium with (EuE) or without (Cont) endometriosis and ectopic lesions (EcE). Based on the Shapiro-Wilk normality test, 1-way ANOVA followed by Tukey’s multiple-comparison test was used to analyze differences among groups (*n* = 5). (**B**) Representative immunohistochemical images of neurofilament-positive (NF^+^) cells in cont, EuE, and EcE (*n* = 5/group). The percentage of NF^+^ neurons was quantified using ImageJ (NIH) and compared using 1-way ANOVA followed by Tukey’s multiple-comparison test among groups. Data are shown as the mean ± SEM. *P < 0.05.

We next examined macrophage infiltration (CD68) and innervation (neurofilament) in the ectopic lesions (EcE) and eutopic endometrium with (EuE) or without (control) spontaneous endometriosis in rhesus macaques (Figure 2, A and B). The clinical details of each animal are described in Supplemental Table 1; supplemental material available online with this article; https://doi.org/10.1172/JCI194136DS1 Although there were no differences in CD68^+^ macrophages in the eutopic endometrium with or without endometriosis, significantly elevated CD68^+^ macrophages were observed in the ectopic lesions (Figure 2A). Neurofilament^+^ neurons were detected in eutopic endometrium and ectopic lesions (Figure 2B). More neurofilament^+^ neurons per area were observed in the ectopic lesions than those in the eutopic endometrium with/without endometriosis (Figure 2B). We observed bundles of neurons in 2 of 5 (1 of 5) of the eutopic endometrial tissues with (without) endometriosis, respectively. However, bundled neural fibers were observed in all ectopic lesions (Figure 2B, white squares).

### Endometriosis-associated hyperalgesia by repeated cycles of lesion induction.

**Figure 3:**
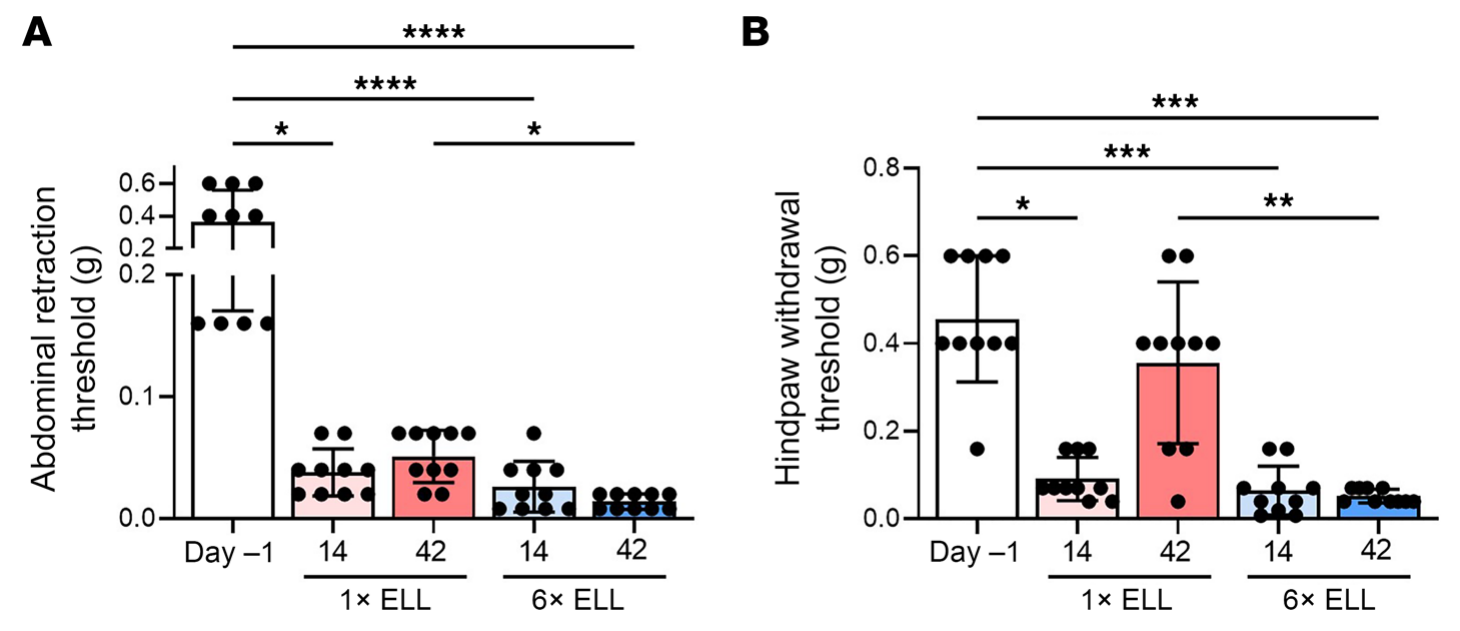
Evaluation of endometriosis-associated hyperalgesia followed by single induction or multiple inductions at 2 or 6 weeks after the last lesion induction. Abdominal (**A**) and hind paw (**B**) withdrawal thresholds were assessed using the von Frey test. Following the Shapiro-Wilk normality test, the Kruskal-Wallis test was used to analyze the differences among the groups. Data are shown as mean ± SEM (*n* = 10). *P < 0.05, **P < 0.01, ***P < 0.001, ****P < 0.0001.

We performed the von Frey test to examine the abdominal and hind paw retraction thresholds in mice and determine whether multiple lesion inductions affect endometriosis-associated hyperalgesia (Figure 3 and Supplemental Figure 1). Both single- and multiple-induction mice withdrew abdominal retraction thresholds with significantly lighter stimuli at 2 or 2 and 6 weeks than preinduction mice (Figure 3A). The multiple inductions showed higher sensitivity than the single induction at 6 weeks (Figure 3A). The hind paw retraction thresholds were more sensitive in the single- and multiple-induction mice at 2 weeks than at the preinduction (Figure 3B). While the sensitivity of hind paw retraction returned to the preinduction level at 6 weeks in the single-induction mice, it remained high in the multiple-induction mice at 6 weeks (Figure 3B).

Additionally, the results of naive controls (a day before the lesion induction, day –1) and sham (multiple PBS injections) showed no differences in sensitivities in the abdomen and hind paw (Supplemental Figure 1). Note: We only showed results for multiple PBS injections at 6 weeks (day 42) in the sham group compared with naive (day –1), as we did not observe any differences in the other sham groups. The results suggest that multiple-induction mice exhibited greater sensitivity not only in the abdomen, where lesions were established, but also at a different body site over extended periods, indicating signs of chronic overlapping pain conditions or widespread pain via central sensitization.

### Activation of microglia and astrocytes in the mouse brain.

**Figure 4:**
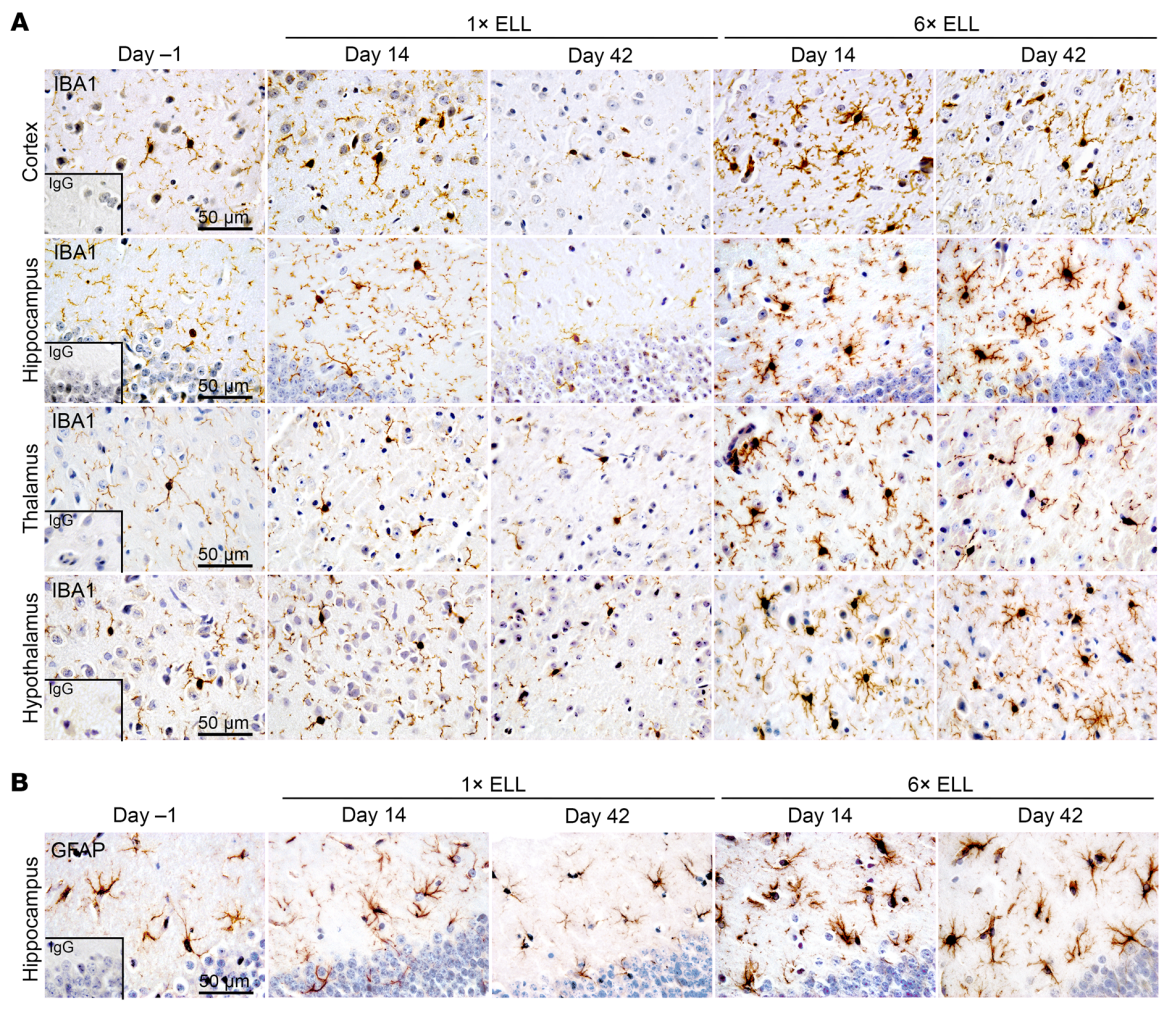
IBA1 and GFAP in the mouse brain. Representative immunohistochemical images of (**A**) IBA1 in the cortex, hippocampus, thalamus, and hypothalamus and (**B**) GFAP in the hippocampus in single- and multiple-induction mice at 2 or 6 weeks after the last lesion induction.

**Figure 5:**
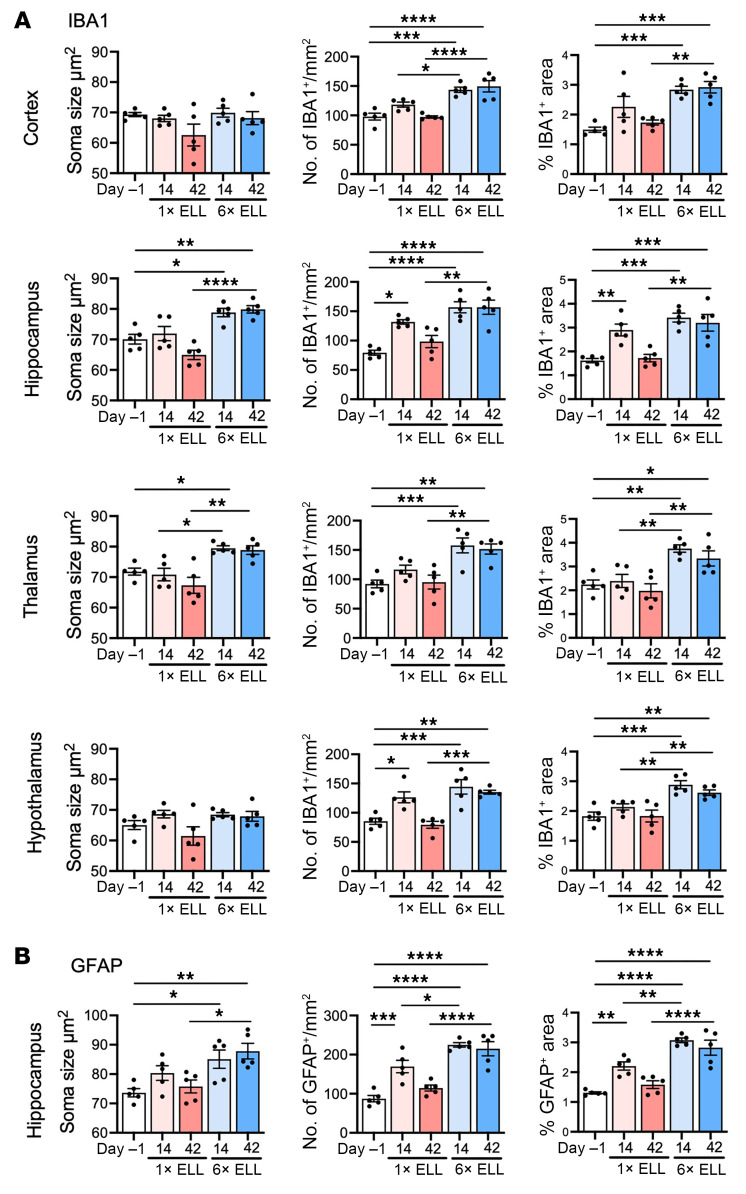
IBA1 and GFAP in the mouse brain. Quantification of immunohistochemical images of (**A**) IBA1 in the cortex, hippocampus, thalamus, and hypothalamus and (**B**) GFAP in the hippocampus in single- and multiple-induction mice at 2 or 6 weeks after the last lesion induction. Following the Shapiro-Wilk normality test, 1-way ANOVA followed by Tukey’s multiple-comparison test was used to analyze differences among groups. Data are shown as mean ± SEM (*n* = 5). *P < 0.05, **P < 0.01, ***P < 0.001, ****P < 0.0001.

Endometriosis-associated pain is maintained in part by central sensitization, which is also driven by neuroinflammation characterized by microglial and astrocytic activation (12, 34–36). Thus, we next analyzed ionized calcium-binding adaptor molecule 1 (IBA1; a marker of microglia) and glial fibrillary acidic protein (GFAP; a marker of astrocytes) in the brain (Figures 4 and 5 and Supplemental Figures 2 and 4). The regions of the mouse brain were selected as the prefrontal cortex for pain processing (37); the hippocampus for pain memory, depression, and anxiety (38, 39); the thalamus for pain modulation and relaying signals (40); and the hypothalamus for mood disorders, stress control, and reproductive function (41).

An increase in microglial soma size is considered a key indicator of microglial activation (42, 43). We thus analyzed soma size, cell number, and percentage of cell-extended area in IBA1^+^ microglia, as previously shown (44). There were no differences in soma size of the microglia within the cortex, hippocampus, thalamus, or hypothalamus of single-induction mice at 2 and 6 weeks (Figure 4A and Figure 5A). In contrast, the microglia of multiple-induction mice had significantly enlarged somas in the hippocampus at 2 and 6 weeks and in the thalamus at 2 weeks compared with those in preinduction mice (Figure 4A and Figure 5A). Soma size in the hippocampus or thalamus of multiple-induction mice at 6 weeks or 2 and 6 weeks, respectively, was greater than that of single-induction mice at these same time points (Figure 4A and Figure 5A). IBA1^+^ microglia number and/or percentage of area were increased in the hippocampus and/or hypothalamus of single-induction mice only at 2 weeks. However, they were elevated in the cortex, hippocampus, thalamus, and hypothalamus of multiple-induction mice at both 2 and 6 weeks (Figure 4A and Figure 5A). Furthermore, multiple inductions yielded more IBA1^+^ microglia than single induction in most brain regions, with some differences at 2 weeks and all at 6 weeks (Figure 4A and Figure 5A).

Astrocyte-mediated neuroinflammation is also a key mechanism underlying chronic pain (12, 45, 46). Chronic neuropathic pain is known to induce astrocyte swelling (47). Thus, we next analyzed astrocytes in brain regions (Figure 4B, Figure 5B, and Supplemental Figure 2, A and B), using the same evaluation methods as for microglia. In the hippocampus, astrocyte soma size was larger in the multiple-induction mice than in preinduction mice at 2 and 6 weeks but was unchanged in the single-induction mice (Figure 4B and Figure 5B). At 6 weeks, astrocyte soma size was greater in the multiple-induction mice than in the single-induction mice (Figure 4B and Figure 5B). GFAP^+^ astrocyte number and percentage of area were elevated in the single-induction mice at 2 weeks and in the multiple-induction mice at 2 and 6 weeks compared with those at preinduction. Multiple inductions further increased the GFAP^+^ astrocyte number and percentage of area than single induction at both time points (Figure 4B and Figure 5B). In contrast, the soma size of the astrocytes did not alter in the cortex, thalamus, and hypothalamus following single or multiple lesion inductions (Supplemental Figure 2, A and B). GFAP^+^ astrocyte number and percentage of area were elevated in the hypothalamus of multiple-induction mice at 2 and 6 weeks (Supplemental Figure 2, A and B). The results of naive and sham controls (multiple PBS injections) did not show any differences in IBA1 and GFAP expression in the cortex, hippocampus, thalamus, and hypothalamus of the mouse brain (Supplemental Figure 4A).

### Activation of microglia and astrocytes in the macaque brain.

**Figure 6:**
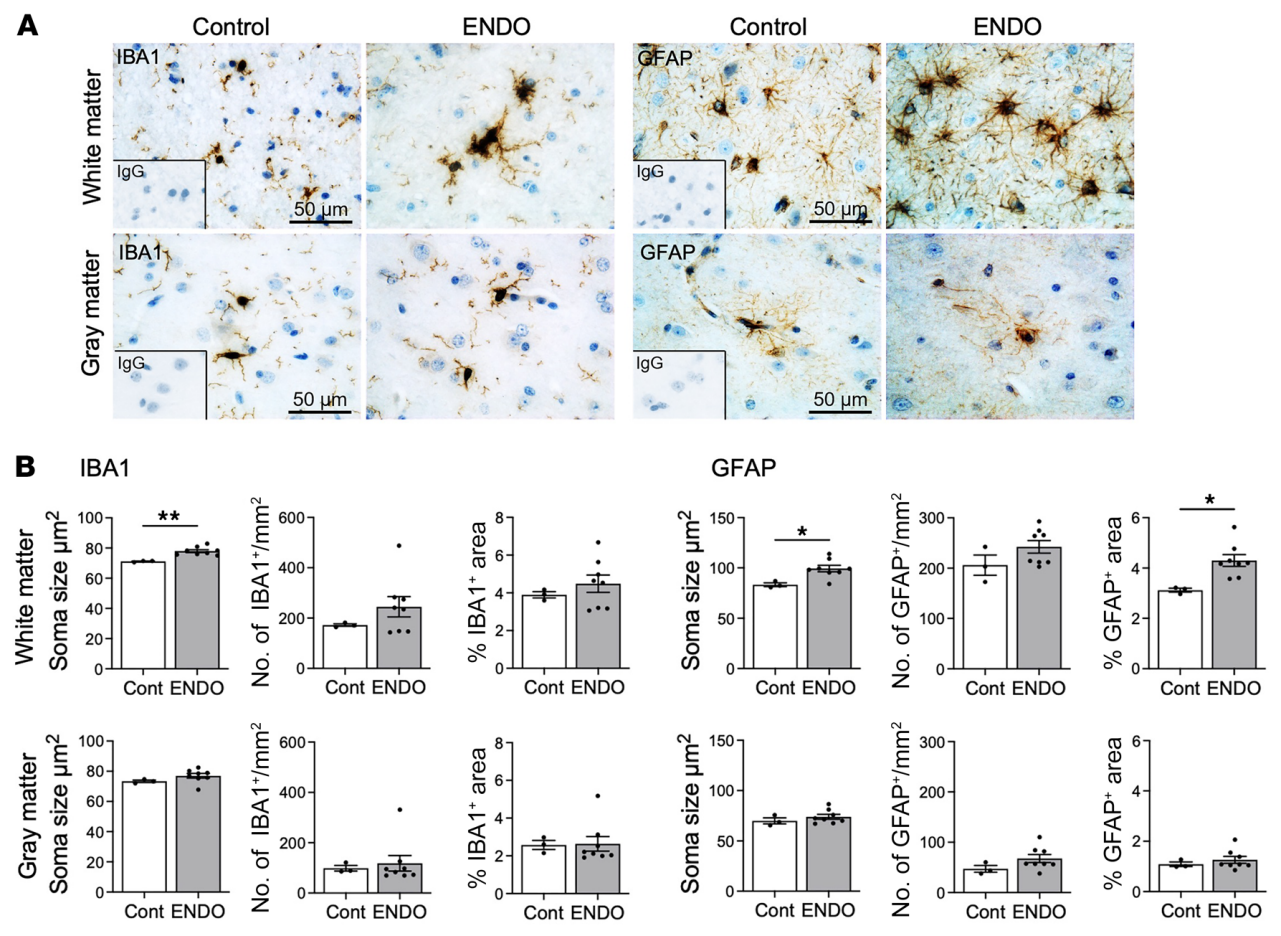
IBA1 and GFAP in the rhesus macaque brain. Representative immunohistochemical images (**A**) and quantification (**B**) of IBA1 and GFAP in the white and gray matter of the prefrontal cortex in rhesus macaques. Following the Shapiro-Wilk normality test, an unpaired 2-tailed *t* test or Mann-Whitney *U* test was used to analyze the differences. Data are shown as mean ± SEM (control: *n* = 3, endometriosis: *n* = 8). *P < 0.05, **P < 0.01.

In the macaque prefrontal cortex, we examined white and gray matter separately, as white matter can predict pain persistence and transition to chronic pain (48). The soma size of IBA^+^ microglia was elevated in the white matter but not in the gray matter in macaques with endometriosis, though IBA1^+^ microglia number and percentage of area were not altered in white and gray matter (Figure 6, A and B). The soma size and percentage of the area of GFAP^+^ astrocytes were increased in the white matter of the prefrontal cortex in macaques with endometriosis, whereas no differences in GFAP^+^ astrocytes were observed in the gray matter (Figure 6, A and B).

### Activation of microglia and astrocytes in the mouse spinal cord.

In the mouse spinal cord, the soma size of microglia and astrocytes was not altered by lesion induction (Supplemental Figure 3, A–C). Multiple inductions induced more IBA1^+^ microglia number and percentage of area compared with those in preinduction mice, whereas single induction only increased percentage of IBA1^+^ area at 2 weeks (Supplemental Figure 3, A and B). GFAP^+^ astrocyte number was also elevated in the spinal cord by multiple inductions at 2 and 6 weeks, and the number was higher in the multiple-induction mice than in the single-induction mice at 6 weeks (Supplemental Figure 3, A and C). In addition, the results of naive and sham (multiple PBS injections) controls did not show any difference in IBA and GFAP expression in the mouse spinal cord (Supplemental Figure 4B).

### Pain-related mediators in the DRG.

**Figure 7:**
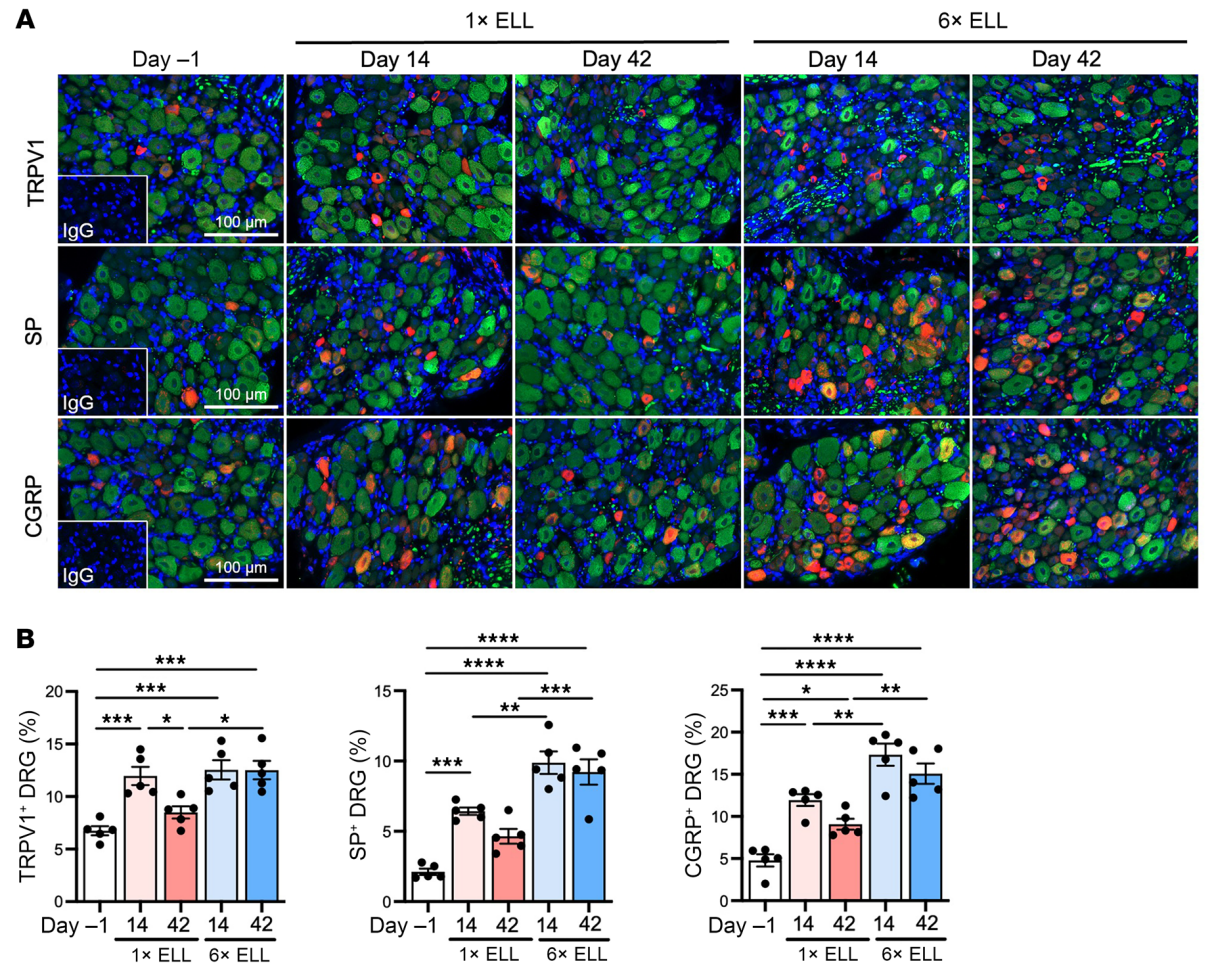
Expression of TRPV1, SP, and CGRP in DRG in single- or multiple-induction mice at 2 or 6 weeks after the last lesion induction. (**A**) Representative images showing DRG sections double-stained with TRPV1, SP, or CGRP (red), and neurofilament (green), as a marker of neural cells. (**B**) Quantification of TRPV1^+^, SP^+^, or CGRP^+^ cells in neurofilament-positive cells. Following the Shapiro-Wilk normality test, 1-way ANOVA followed by Tukey’s multiple-comparison test was used to analyze differences among groups. Data are shown as the mean ± SEM (*n* = 5). *P < 0.05, **P < 0.01, ***P < 0.001, ****P < 0.0001.

DRG are sensory neurons that detect and transmit stimuli to the CNS (49). We have reported increased expression of transient receptor potential channels, TRPV1, and neurotransmitters, such as SP and CGRP, in mouse endometriosis (25). We thus examined TRPV1, SP, and CGRP in the bilateral lumbar DRG (L4–6), the primary spinal ganglia receiving sensory input from pelvic organs (Figure 7). Both single and multiple inductions increased TRPV1, SP, and CGRP expression at 2 weeks compared with preinduction levels (Figure 7, A and B). Elevated TRPV1^+^ and SP^+^ DRG remained high in the multiple-induction mice at 6 weeks but not in the single-induction mice, while CGRP^+^ DRG were still high in the single-induction mice at 6 weeks (Figure 7, A and B). Furthermore, more SP^+^ and CGRP^+^ DRG were detected in the multiple-induction mice than in the single-induction mice at 2 and 6 weeks (Figure 7, A and B). These results indicate that multiple inductions induce prolonged stimulation of nociceptor neurons in the DRG. We did not observe any different TRPV1, SP, and CGRP expression in the mouse DRG between naive and sham (multiple PBS injections) controls (Supplemental Figure 4C).

### Macrophage dynamics in the peritoneal cavity.

**Figure 8:**
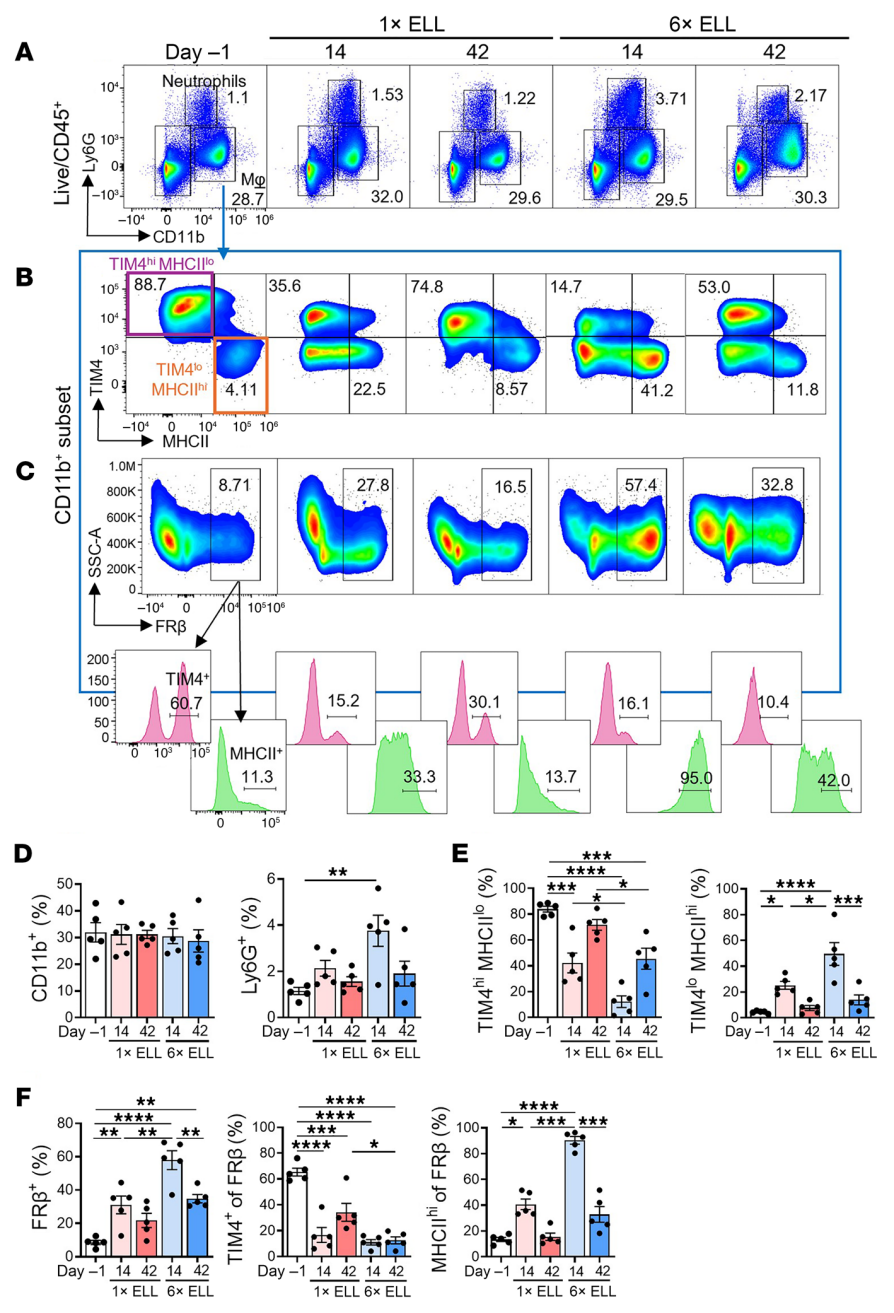
Comparison of peritoneal immune cell profiles in single- and multiple-induction mice at 2 or 6 weeks after the last lesion induction. (**A**) Representative flow plots illustrating the composition of CD11b^+^ and Ly6G^+^ cells. (**B**) CD11b^+^ cells were further gated by TIM4 and MHCII. (**C**) CD11b^+^ cells were further gated by FRβ (top), and FRβ^+^ cells were then gated by TIM4 and MHCII (bottom). Proportions of CD11b^+^ or Ly6G^+^ (**D**) and TIM4^hi^MHCII^lo^ and TIM4^lo^MHCII^hi^ (**E**) are shown. (**F**) Proportions of FRβ^+^ of CD11b^+^ cells and TIM4^+^ or MHCII^hi^ of FRβ^+^ macrophages were shown. Following the Shapiro-Wilk normality test, the differences in MHCII^hi^ in FRβ^+^ macrophages were analyzed with the Kruskal-Wallis test; all comparisons among groups were performed using 1-way ANOVA followed by Tukey’s multiple-comparison test. Data are shown as the mean ± SEM (*n* = 5). *P < 0.05, **P < 0.01, ***P < 0.001, ****P < 0.0001.

Heterogeneous macrophage populations time-dependently alter in the peritoneum after lesion induction in mice (25). We next examined how multiple inductions affect pro-inflammatory macrophages (T cell immunoglobulin and mucin domain containing 4–low, TIM4^lo^; major histocompatibility complex II–high, MHCII^hi^), folate receptor β–positive (FRβ)^+^ macrophages, and residential macrophages (TIM4^hi^MHCII^lo^), as well as neutrophils (lymphocyte antigen 6G, Ly6G^+^) (Figure 8). First, we confirmed that naive and sham (multiple PBS injections) controls did not show any altered immune cell profiles in the peritoneal cavity (Supplemental Figure 5). Although there were no significant differences in the integrin subunit α M (ITGAM, CD11b)^+^ total macrophage population between single and multiple inductions at 2 and 6 weeks, Ly6G^+^ neutrophils were significantly elevated in the multiple-induction mice at 2 weeks (Figure 8, A and D). CD11b^+^ macrophages were further gated to TIM4^lo^MHCII^hi^ and TIM4^hi^MHCII^lo^ macrophages to examine pro-inflammatory and residential macrophages, respectively (Figure 8B). Both single and multiple inductions reduced TIM4^hi^MHCII^lo^ macrophages at 2 weeks as a sign of macrophage disappearance reaction (MDR). The population of TIM4^hi^MHCII^lo^ macrophages at 2 weeks was lower in the multiple-induction mice than in the single-induction mice (Figure 8, B and E), suggesting that the multiple inductions induced severe MDR. At 6 weeks, residential macrophages in the single-induction mice returned to preinduction levels, whereas they remained lower in the multiple-induction mice. Thus, the MDR induced by the single induction was replenished and recovered, but the MDR induced by multiple inductions was not entirely resolved at 6 weeks (Figure 8, B and E). Single and multiple inductions elevated TIM4^lo^MHCII^hi^ pro-inflammatory macrophages at 2 weeks, with the latter further increasing their population (Figure 8, B and E). TIM4^lo^MHCII^hi^ macrophages returned to preinduction levels in both groups at 6 weeks (Figure 8, B and E). We previously reported a FRβ^+^ macrophage population differentiated from monocyte-derived pro-inflammatory macrophages and possessing residential macrophage characteristics (50). Single and multiple inductions increased FRβ^+^ macrophages at 2 weeks compared with preinduction levels (Figure 8, C and F). FRβ^+^ macrophages were higher in the multiple-induction mice than in the single-induction mice at 2 weeks (Figure 8, C and F). High levels of FRβ^+^ macrophages were sustained at 6 weeks in the multiple-induction mice (Figure 8, C and F). When FRβ^+^ macrophages were further gated to TIM4^+^ or MHCII^hi^, most of the FRβ^+^ macrophages expressed high MHCII but limited TIM4 after lesion induction (Figure 8, C and F). Specifically, MHCII^hi^FRβ^+^ macrophages were significantly elevated following multiple inductions at 2 weeks (Figure 8, C and F). These results suggest that elevated FRβ^+^ macrophages after lesion induction were newly recruited, monocyte-derived, highly inflammatory macrophages and that multiple inductions further recruited and elevated them in the peritoneal cavity.

### B and T cell dynamics in the peritoneal cavity.

In addition to macrophages, we examined peritoneal B and T cells (Supplemental Figure 6). B lymphocyte antigen CD19 (CD19^+^) B cells were reduced in the multiple-induction mice at 2 weeks compared with those in the preinduction mice (Supplemental Figure 6, A and B). CD3^+^ T cells were elevated at 2 weeks in the multiple-induction mice following increased cytotoxic/killer T cells (CD8^+^) and T helper cells (CD4^+^) (Supplemental Figure 6, A and B). CD4^+^ T cells were higher at 6 weeks in the multiple-induction mice than in the single-induction mice (Supplemental Figure 6, A and B).

### Inflammatory environment establishment in the peritoneal cavity.

**Figure 9:**
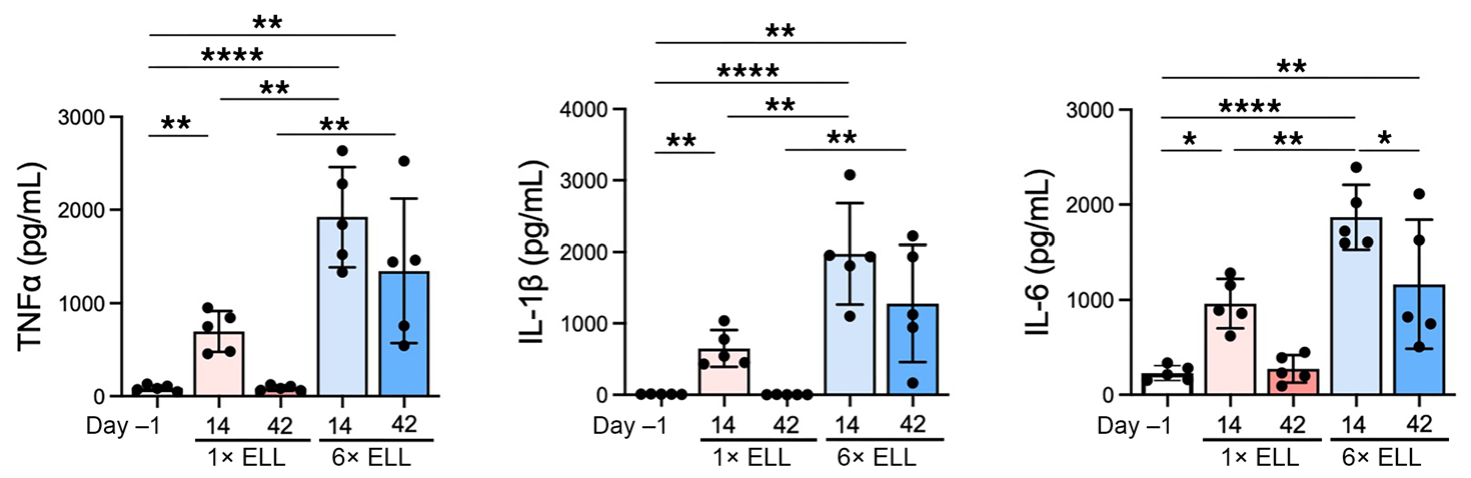
Pro-inflammatory cytokine levels (TNF-α, IL-1β, and IL-6) in the peritoneal fluid analyzed by IQELISA. Following the Shapiro-Wilk normality test, 1-way ANOVA followed by Tukey’s multiple-comparison test was used to analyze differences among groups. Data are shown as the mean ± SEM (*n* = 5). *P < 0.05, **P < 0.01, ****P < 0.0001.

To confirm whether multiple inductions elevate peripheral inflammation, peritoneal TNF-α, IL-1β, and IL-6 protein concentrations were assessed (Figure 9), as these cytokines are considered key factors in maintaining the aberrant peritoneal inflammatory environment, promoting lesion growth, and mediating peripheral sensitization (51–53). Single and multiple inductions significantly elevated secreted TNF-α, IL-1β, and IL-6 levels in the peritoneal cavity at 2 weeks (Figure 9). All cytokine levels were higher in the multiple-induction mice than in the single-induction mice at 2 weeks (Figure 9). Elevated cytokine levels returned to the preinduction levels in the single-induction mice at 6 weeks; however, they remained high in the multiple-induction mice (Figure 9). These results further support that the multiple inductions establish an aberrant chronic inflammatory environment in the peritoneal cavity.

### Dienogest and fingolimod did not affect lesion progression.

**Figure 10:**
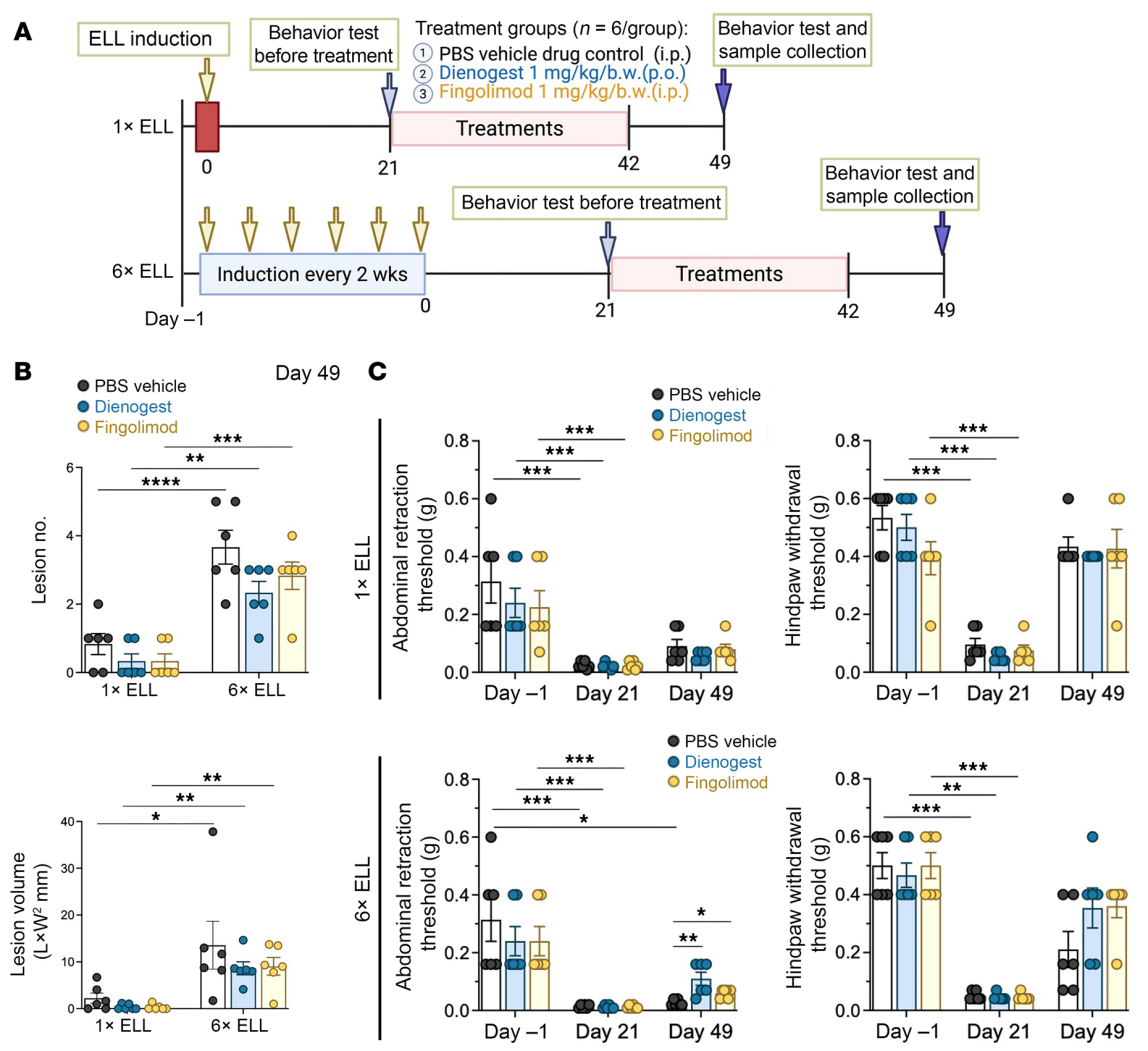
Evaluation of the effects of dienogest and fingolimod on endometriosis-like lesions and hyperalgesia using a single or multiple lesion induction mouse model of endometriosis. (**A**) Experimental design for study 2 as described in Methods. (**B**) Comparison of lesion numbers and size in single- or multiple-induction mice at 7 weeks (day 49) after the last lesion induction between the PBS vehicle (drug control in animals with induced disease) and treatment groups (*n* = 6; animals without lesions in the single-induction group were included for all further analysis in study 2). If >1 lesion was observed, the average lesion size per animal was used for the analysis. (**C**) Evaluation of the effects of dienogest and fingolimod on endometriosis-associated abdominal and hind paw hyperalgesia by the von Frey test (*n* = 6/group). The group differences in lesion numbers and sizes were assessed using the Kruskal-Wallis test. For behavioral analysis, time-dependent differences within a group were assessed using the Kruskal-Wallis test comparing thresholds at different time points with those on day –1. To compare the effects of dienogest and fingolimod at each time point, the Kruskal-Wallis test was used to assess the differences among groups. Data are shown as the mean ± SEM. *P < 0.05, **P < 0.01, ***P < 0.001, ****P < 0.0001.

Since multiple lesion induction induces further elevated hyperalgesia and glial activation, we next examined whether targeting neuroinflammation, compared with a current clinically approved endometriosis treatment, could improve hyperalgesia in our model. We chose fingolimod (FTY720, an sphingosine 1-phosphate receptor modulator and immunosuppressor for multiple sclerosis) (54, 55) for its efficacy in lesion-induced hyperalgesia and neuroinflammation in our models, as it reduces neuropathic pain behavior, central sensitization, and neuroinflammation (56, 57). Based on previously published mouse studies (56, 57), a dose of 1 mg/kg/body weight (b.w.), administered i.p., was selected for our study. We also chose dienogest, a synthetic progestin with antiestrogenic effects, which is administered orally for endometriosis and is known to reduce endometriosis-associated pain (58, 59). Based on previous studies in the mouse model of endometriosis (60, 61), a dose of 1 mg/kg/b.w. oral administration was selected for this study. The integration of these treatments with the multiple-induction model is described in study 2 of the Methods section and summarized in Figure 10A. Treatments with dienogest or fingolimod did not significantly impact lesion numbers (Figure 10B), which were similarly increased following multiple rounds of lesion induction (Figure 1B and Figure 10B). Further, the relative size of lesions was not impacted by either drug. However, dienogest reduced CD68^+^ macrophages compared with those in the PBS vehicle control group (Supplemental Figure 7, A and B). Both dienogest and fingolimod decreased LYVE1^+^ cells, whereas PGP9.5^+^ nerve cells were not affected by dienogest or fingolimod (Supplemental Figure 7, A and B).

### Dienogest and fingolimod alleviate endometriosis-associated hyperalgesia.

We next performed the von Frey test to determine whether dienogest and fingolimod improve endometriosis-associated hyperalgesia (Figure 10C). In the single-induction group, mice showed greater sensitivity in both the abdomen and hind paws at 3 weeks (day 21) before treatments, as expected. In support of the results at 6 weeks (Figure 3), both sensitivities at 7 weeks (day 49) were no longer significantly different from those of preinduction mice. Dienogest and fingolimod did not improve the sensitivity of either the abdomen or the hind paws in the single-induction group (Figure 10C). In the multiple-induction group, the abdominal retraction threshold at 7 weeks remained significantly more sensitive than on day –1 in the PBS control mice (Figure 10C). The abdominal sensitivity at 7 weeks in the PBS control group induced by multiple inductions was greater than that of the single induction (Supplemental Figure 8A). Dienogest and fingolimod improved abdominal sensitivity at 7 weeks (Figure 10C). Both treatments tended to improve hind paw retraction thresholds at 7 weeks, but no significant improvement was observed (Figure 10C). The PBS control group returned to the preinduction level at 7 weeks, which may have prevented differences. Compared with the single induction, multiple inductions induced greater hind paw sensitivity at 7 weeks in the PBS control mice but not in the dienogest and fingolimod treatment groups (Supplemental Figure 8B). These results may reflect ongoing improvements in mice that received dienogest and fingolimod treatments.

### Dienogest and fingolimod reduce microglial and astrocytic activation in the mouse brain.

**Figure 11:**
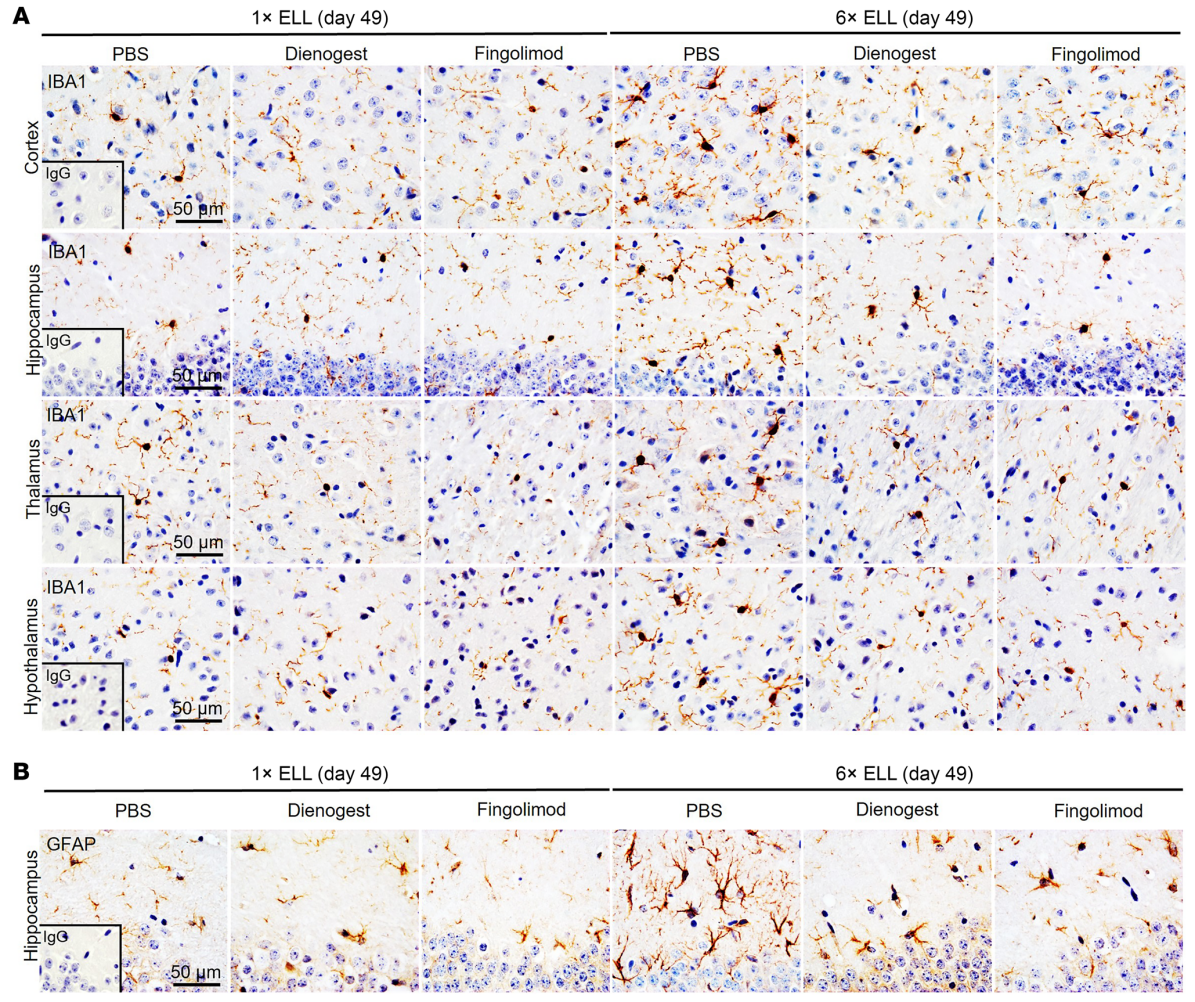
IBA1 and GFAP in the mouse brain. Representative immunohistochemical images of (**A**) IBA1 in the cortex, hippocampus, thalamus, and hypothalamus and (**B**) GFAP in the hippocampus in single- and multiple-induction mice followed by dienogest or fingolimod treatment.

**Figure 12:**
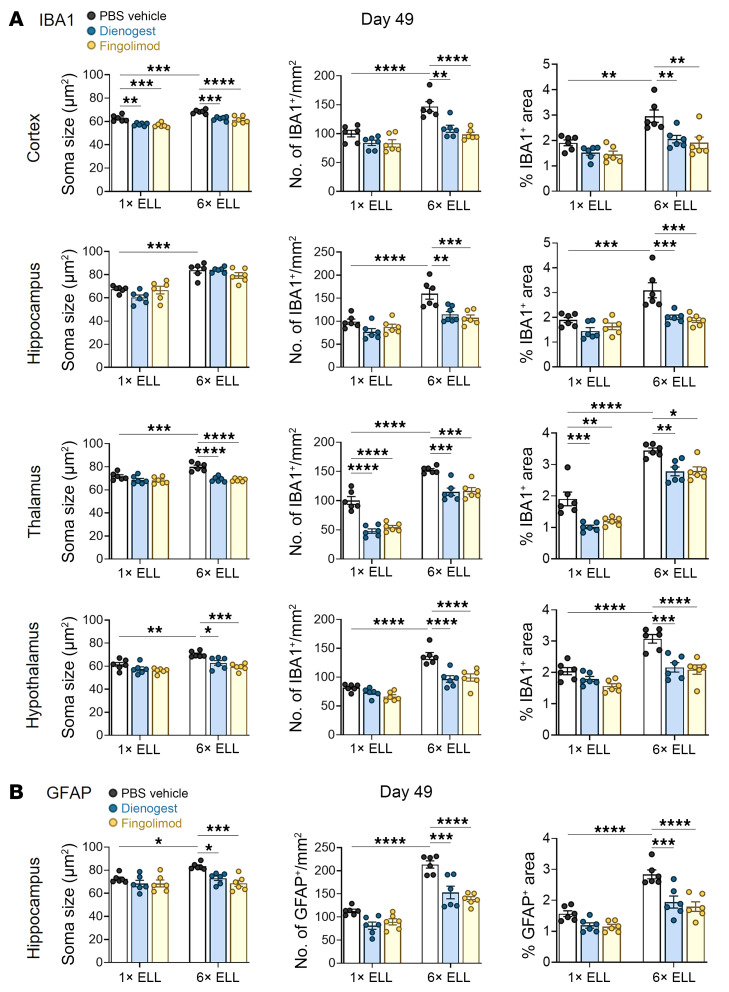
IBA1 and GFAP in the mouse brain. Quantification of immunohistochemical images of (**A**) IBA1 in the cortex, hippocampus, thalamus, and hypothalamus and (**B**) GFAP in the hippocampus in the single- or multiple-induction mice, followed by dienogest or fingolimod treatment. Following the Shapiro-Wilk normality test, 1-way ANOVA followed by Tukey’s multiple-comparison test was used to analyze differences among groups. Data are shown as mean ± SEM (*n* = 6). *P < 0.05, **P < 0.01, ***P < 0.001, ****P < 0.0001.

We next examined whether dienogest and fingolimod reduce glial activation induced by single or multiple lesion induction (Figures 11 and 12). In the cortex, hippocampus, thalamus, and hypothalamus, soma size, cell number, and percentage of area occupied by IBA1^+^ microglia were increased by multiple inductions in the PBS control group at 7 weeks (day 49). However, the soma sizes of IBA1^+^ microglia in the cortex and hypothalamus did not differ significantly at 6 weeks (day 42) between the single- and multiple-induction groups (Figure 5A). Dienogest and fingolimod were effective in reducing IBA1^+^ microglial soma size, cell number, and/or percentage of area in most brain regions (Figure 11A and Figure 12A). Interestingly, reductions by dienogest and fingolimod were observed across all brain regions with 3 parameters (size, number, and percentage area) in the multiple-induction group, except for soma size in the hippocampus (Figure 11A and Figure 12A). However, their effects in the single-induction group were limited to soma size in the cortex and to cell number and percentage of cell-extended area in the thalamus.

We observed that soma size, cell number, and/or percentage of area of GFAP^+^ astrocytes were elevated by multiple inductions in the PBS control group in the hippocampus and hypothalamus (Figure 11B, Figure 12B, and Supplemental Figure 9, A and B), in agreement with findings in Figure 4B, Figure 5B, and Supplemental Figure 2, A and B. We also realized that multiple inductions elevated the percentage of GFAP^+^ area in the thalamus of the PBS control group at 7 weeks (Supplemental Figure 9, A and B). Dienogest and fingolimod reduced GFAP^+^ soma size, cell number, and percentage of cell-extended area in the hippocampus but only in the multiple-induction group (Figure 11B and Figure 12B). Both treatments also reduced GFAP^+^ cell number and the percentage of area in the hypothalamus, which were elevated by the multiple inductions (Supplemental Figure 9, A and B). The inhibitory effects of dienogest and fingolimod on microglial and astrocyte-mediated neuroinflammation correlated with their effects on abdominal hyperalgesia (Figure 10C). Thus, the results support that neuroinflammation is a part of endometriosis-associated hyperalgesia.

### Dienogest and fingolimod reduce neural sensitization in the DRG but not glial activation in the spinal cord.

In addition to IBA1^+^ microglia and GFAP^+^ astrocytes in the brain, we examined their activities in the spinal cord following dienogest and fingolimod treatment (Supplemental Figure 10, A–C). However, dienogest and fingolimod did not alter any parameters in IBA1^+^ microglia and GFAP^+^ astrocytes in the spinal cord.

**Figure 13:**
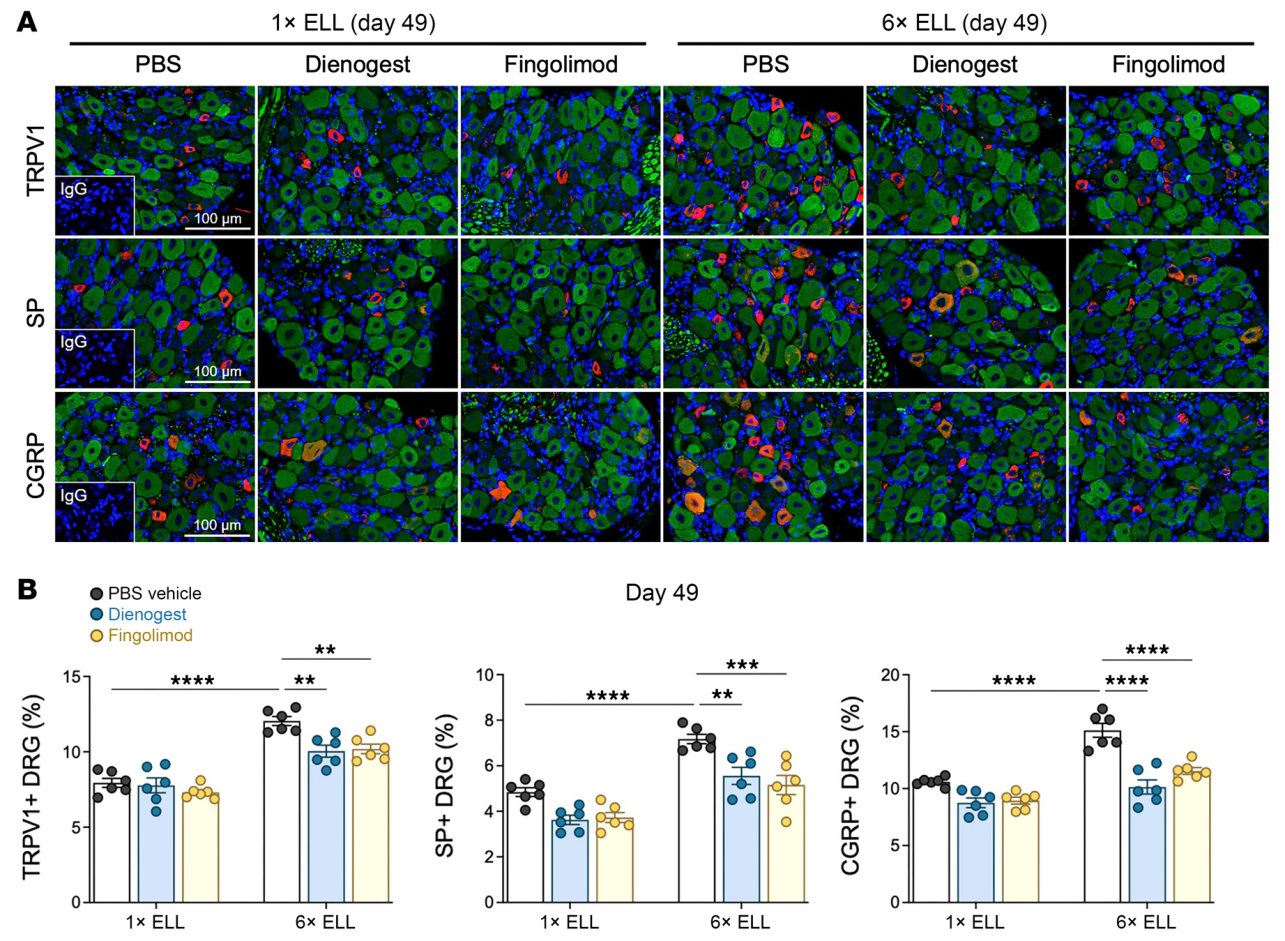
Expression of TRPV1, SP, and CGRP in DRG in the single- or multiple-induction mice followed by dienogest or fingolimod treatment. (**A**) Representative images showing DRG sections double-stained with TRPV1, SP, or CGRP (red), and neurofilament (green), as a marker of neural cells. (**B**) Quantification of TRPV1^+^, SP^+^, or CGRP^+^ cells in neurofilament-positive cells. Following the Shapiro-Wilk normality test, 1-way ANOVA followed by Tukey’s multiple-comparison test was used to analyze differences among groups. Data are shown as the mean ± SEM (*n* = 6). **P < 0.01, ***P < 0.001, ****P < 0.0001.

In the DRG, we confirmed that multiple inductions elevated TRPV1^+^, SP^+^, and CGRP^+^ DRG in the PBS control group at 7 weeks (see Figures 7 and 13). Dienogest and fingolimod reduced elevated TRPV1^+^, SP^+^, and CGRP^+^ DRG in the multiple-induction group but not in the single-induction group (Figure 13, A and B). The results also align with the behavioral results in Figure 10C and Supplemental Figure 8.

### Impact of dienogest and fingolimod on peritoneal immune cells.

In the peritoneal immune cell profiles, dienogest and fingolimod did not alter total macrophages (CD11b^+^) and neutrophils (Ly6G^+^) (Supplemental Figure 11, A and B). TIM4^hi^MHCII^lo^ macrophages were lower, and FRβ^+^ macrophages were higher in the PBS group with multiple inductions at 7 weeks (Supplemental Figure 11, C and D), as confirmed by the results in Figure 8. Dienogest reduced FRβ^+^ macrophages in the multiple-induction group (Supplemental Figure 11C), whereas TIM4^hi^MHCII^lo^ and TIM4^lo^MHCII^hi^ macrophages were not improved by dienogest (Supplemental Figure 11, D and E). In contrast, fingolimod reduced TIM4^hi^MHCII^lo^ macrophages in the single-induction group compared with the PBS group (sustained MDR at 7 weeks) and elevated TIM4^lo^MHCII^hi^ and FRβ^+^ macrophages (still maintained high pro-inflammatory macrophages) in the single-induction group (Supplemental Figure 11, C–E). The results are likely due to the daily i.p. administration of fingolimod for 3 weeks. Fingolimod itself was somewhat sensitive to macrophage differentiation in the single-induction group, though fingolimod’s effect was only observed in the macrophage differentiation and replenishment. However, in the multiple-induction group, fingolimod improved elevated FRβ^+^ macrophages at 7 weeks, similar to the effect of the dienogest treatment (Supplemental Figure 11C). These results indicate that peritoneal macrophage differentiation is more sensitive to the route of drug administration, such as multiple i.p. injections. Dienogest and fingolimod attenuated elevated T cell (CD3^+^, CD8^+^, and CD4^+^) but did not affect B cell (CD19^+^) profiles in the multiple-induction group (Supplemental Figure 11, F–I).

### Dienogest and fingolimod did not alter cytokine levels in the peritoneal cavity.

Confirming the results of peritoneal cytokine levels in Figure 9, multiple inductions in the PBS group elevated TNF-α and IL-1β, but not IL-6, compared with those in single induction on day 49 (Supplemental Figure 12). However, dienogest and fingolimod did not reduce elevated cytokine levels in the peritoneal cavity (Supplemental Figure 12), indicating that at least these cytokines are not significant factors targeted by dienogest and fingolimod in reducing glial activation and peripheral sensitization.

## Discussion

### The multiple-induction model improves our understanding of endometriosis-associated CPP.

Approximately 60%–80% of women with endometriosis suffer endometriosis-associated CPP (62, 63), which is 13 times higher than in endometriosis-free patients (63). Women with endometriosis experience menstrual cyclic and acyclic pain, i.e., dysmenorrhea with dyschezia, dysuria, or dyspareunia (62), and pain can be expanded throughout the pelvis and abdomen and further referred to the back and legs (62). Women with endometriosis are often diagnosed with bladder and colon sensory dysfunctions, such as inflammatory bowel disease or overactive bladder syndrome (64). Widespread pain is also a common experience in women with endometriosis. Phan et al. (65) have reported that endometriosis-associated CPP often causes myofascial dysfunction and sensitization beyond the pelvic regions that may be initiated or maintained by ongoing pelvic floor spasms. These comorbidities indicate a wide range of endometriosis-associated CPP and a more complex pathophysiology of endometriosis. Recent evidence suggests that protracted peripheral and central sensitization are present in endometriosis patients with CPP (11). Neuroinflammation can drive endometriosis-associated CPP via chronic inflammation and central sensitization (44, 66–68). As menstrual cycles repeatedly occur in women, we designed the present study to induce multiple endometrial inoculations to mimic recurrent retrograde menstruation and to understand endometriosis-associated CPP via peripheral chronic inflammation, neuroinflammation, and central sensitization. Although inoculation of progesterone-withdrawn “mense-like” endometrial tissues is a viable strategy to induce lesions (69–72), we have confirmed that inoculations of PMSG-primed/synchronized endometrial tissues also develop endometriosis-like lesions, hyperalgesia, peripheral inflammation, and immune cell profiles similar to those in the “mense-like” model, and we chose them as donor endometrial tissues in the present study (24, 25, 50). As an important phenotypic finding, multiple lesion inductions resulted in greater hyperalgesia, with prolonged hind paw sensitization and increased abdominal sensitivity. While abdominal sensitivity is considered peripheral visceral pain due to thinner skin and less underlying muscle, the hind paw can be affected by both peripheral and central sensitization, which involve neural pathways (73). Although the lesion numbers increased with multiple inductions as a nature of the mouse model of endometriosis (~80% of mice develop lesions), endometriosis-associated pain is not correlated with disease extent in women with endometriosis (11). Thus, endometriotic lesion–dependent pain is apparent; however, the lesions cannot be the sole source of endometriosis-associated CPP. Because the multiple-induction model induces elevated, prolonged hyperalgesia, we used it to understand the mechanisms underlying endometriosis-associated CPP.

### Prolonged glial activation leading to central sensitization and endometriosis-associated CPP induced by multiple lesion inductions.

Our results showed prolonged glial activation in several brain regions in multiple-induction mice. A consistent increase in the soma size of IBA^+^ microglia and/or cell number was observed in the brain and spinal cord, which are characteristic indicators of neuroinflammation in the CNS. Interestingly, the larger soma size of microglia and astrocytes with elevated IBA^+^ or GFAP^+^ cells was only observed in the hippocampus. Many studies have reported hippocampus abnormalities in patients experiencing chronic pain, anxiety, and depression (74). GFAP^+^ astrocytes in the hippocampus are associated with mood disorders in persistent pain states (46, 74). Endometriosis is known to affect the mental health and emotional well-being of women, leading to anxiety and depression (75, 76). Due to abundant glial activation in the hippocampus induced by multiple inductions, cyclic sources of peripheral input are likely to induce neuroinflammation for extended periods, causing anxiety and depression and reducing the quality of life in women with endometriosis. IBA1^+^ microglial cells were also increased in the cortex, which has important pain-processing functions connecting stimuli to other brain regions, such as the hippocampus and thalamus (37). As-Sanie et al. (77, 78) demonstrate that changes in regional gray matter volume within the central pain system in the cortex play an important role in the development of endometriosis-associated pain, regardless of the presence of endometriotic lesions. Mansour et al. (48) have reported that white matter abnormalities may predict pain persistence and transition to chronic pain, though gray and white matter play a role in pain processing (37). Our results showed that the size of IBA1^+^ microglia and GFAP^+^ astrocytes was more extensive in the white matter of the prefrontal cortex in macaques with endometriosis. More GFAP^+^ astrocytes were also observed in the white matter. While the connection between neuroinflammation and altered cortical gray matter volume remains unclear, changes in the central pain system are crucial to the development of endometriosis-associated CPP, and white matter neuroinflammation may be intensely involved in sustaining CPP. In support of this, central sensitization and pain-related behaviors are positively correlated in naturally occurring endometriosis macaques (32).

In agreement with the findings of our study, neuroinflammation associated with endometriosis has been reported in a mouse study (44). Bashir et al. (44) showed that lesion-induced mice experienced increased pain and discomfort, as well as hyperalgesia on day 15 after lesion induction. Increased soma size of microglia was also observed in the cortex, hippocampus, thalamus, and hypothalamus in a mouse model of endometriosis following a single-lesion induction (44). Their results further indicate that 2 weeks after lesion induction is a critical time for the establishment of neuroinflammation. Additionally, Li et al. (79) demonstrated that hyperalgesia, anxiety, and depression-related behavior were observed in the lesion-induced mice with altered synaptic transmission in glutamatergic and GABAergic synapses in the amygdala. Dodds et al. (80) also showed that adaptations in central glial reactivity were attributed to the presence of lesions in a mouse model of endometriosis. Our results further confirm their findings and suggest that multiple lesion inductions enhance glial activation and prolong neuroinflammation in the nervous system, thereby initiating and driving central sensitization, leading to chronic and widespread endometriosis-associated CPP.

### Peripheral chronic inflammation and immune cell contribution induced by multiple lesion inductions.

In the present study, multiple lesion inductions elevated peripheral inflammation, as evidenced by persistently elevated TNF-α, IL-1β, and IL-6 levels in peritoneal fluid for extended periods. In contrast, single induction increased cytokine levels only up to 2 weeks after lesion induction, suggesting that initial inflammation has probably resolved. The results of immune cell distribution in the peritoneal cavity support the establishment of a chronic inflammatory environment via multiple inductions. Peritoneal macrophages are highly diverse (50, 81), differ in ontogeny (82), and exhibit transcriptional and functional divergence in response to local environmental signals (83). When endometrial tissue is introduced into the peritoneum, an acute inflammatory response occurs. Peritoneal resident macrophages (TIM4^hi^MCHII^lo^) are important for initial uptake, as they adhere to the mesothelium to cover organs (81, 82) or die via pyroptosis, releasing pro-inflammatory cytokines, such as IL-1β (84), called MDR. If residential macrophages die/disappear, they appear to be replaced by bone marrow–/monocyte-derived macrophages (85). Our study showed that MDR induced by multiple inductions was more severe than that induced by a single induction. In support of our previous study (25), MDR was recovered by 6 weeks in the single-induction mice, whereas it was not fully resolved at 6 weeks in the multiple-induction mice. Following MDR results, a more significant monocyte-derived pro-inflammatory macrophage population was found in the multiple-induction mice, indicating higher levels of inflammation and severe macrophage replenishment. Our previous study has demonstrated that monocyte-derived pro-inflammatory macrophages further differentiate into FRβ^+^ macrophages with some resident macrophage features (= large peritoneal macrophages) (50). Herein, we show that newly recruited FRβ^+^ macrophages highly express MHCII but express TIM4 at low levels. These results suggest that repetitive inoculations of endometrial tissues induce persistent inflammatory stimuli that enhance and sustain peripheral chronic inflammation, likely elevating FRβ^+^ macrophages. Because neurotransmitters (SP and CGRP) and TRPV1 were greater in the DRG in the multiple-induction mice, chronic inflammatory stimuli further affect the peripheral sensory nervous system. Of note, the peritoneal T cell population was increased in multiple-induction mice, a finding not observed in our previous study using a single-induction mouse model of endometriosis (24, 25, 86). CD8^+^ T cells have been reported to be enriched in the endometriotic lesions, potentially linked to endometriosis development, infertility, and chronic pain (87, 88). Further involvement of T cell functions and CPP remains to be studied.

### Clinical and translational relevance of targeting neuroinflammation for endometriosis-associated neuroinflammation.

Our study showed that dienogest and fingolimod treatments reduced microglial and astrocyte activation and peripheral sensitization, and improved abdominal hyperalgesia, especially following multiple inductions. Efficacy was limited in the single-induction group, as we assume the mice were less sensitive and almost recovered by 7 weeks after lesion induction. Dienogest is a selective progesterone receptor agonist and has been approved as a daily oral tablet for the treatment of endometriosis and its associated pain in most countries, except in the United States for endometriosis-related pain (58, 59, 89). It has been shown to have antiproliferative/antiestrogenic and antiinflammatory effects on lesions (58). It can also reduce nerve fiber density in the eutopic endometrium in endometriosis (90). Our results support its usefulness in reducing endometriosis-associated CPP. As an additional finding, dienogest is effective in reducing glial and astrocyte activation. However, the underlying mechanism of dienogest’s antiestrogenic, antiinflammatory, or direct action on glial activation and peripheral sensitization in our model remains to be elucidated, as dienogest did not affect lesion progression and growth or peritoneal cytokine levels.

Fingolimod has been used for the treatment of relapsing multiple sclerosis (91, 92), where it antagonizes S1PR1 to inhibit lymphocyte migration into the circulation, thereby reducing systemic inflammation (92). It can also reduce neuropathic pain and S1PR1-dependent central sensitization (56). Rigorous recent studies demonstrate that fingolimod directly affects the CNS by preventing the pro-inflammatory activation of microglia and astrocytes (93, 94). Fingolimod protects the blood-brain barrier (BBB) integrity, inhibiting neuroinflammation and decreasing neuronal apoptosis in the cortex and hippocampus (95). Thus, the present study suggests that targeting microglial and astrocytic activation reduces endometriosis-associated CPP, probably via modulation of central sensitization. The results further indicate that neuroinflammatory mechanisms are crucial to understand endometriosis-associated CPP and central sensitization. However, the use of fingolimod is currently limited to treating relapsing forms of multiple sclerosis. Serious side effects of fingolimod include bradycardia, macular edema, increased infection risk, skin cancer, headache, diarrhea, respiratory issues, and back pain (92).

### Conclusion.

**Figure 14:**
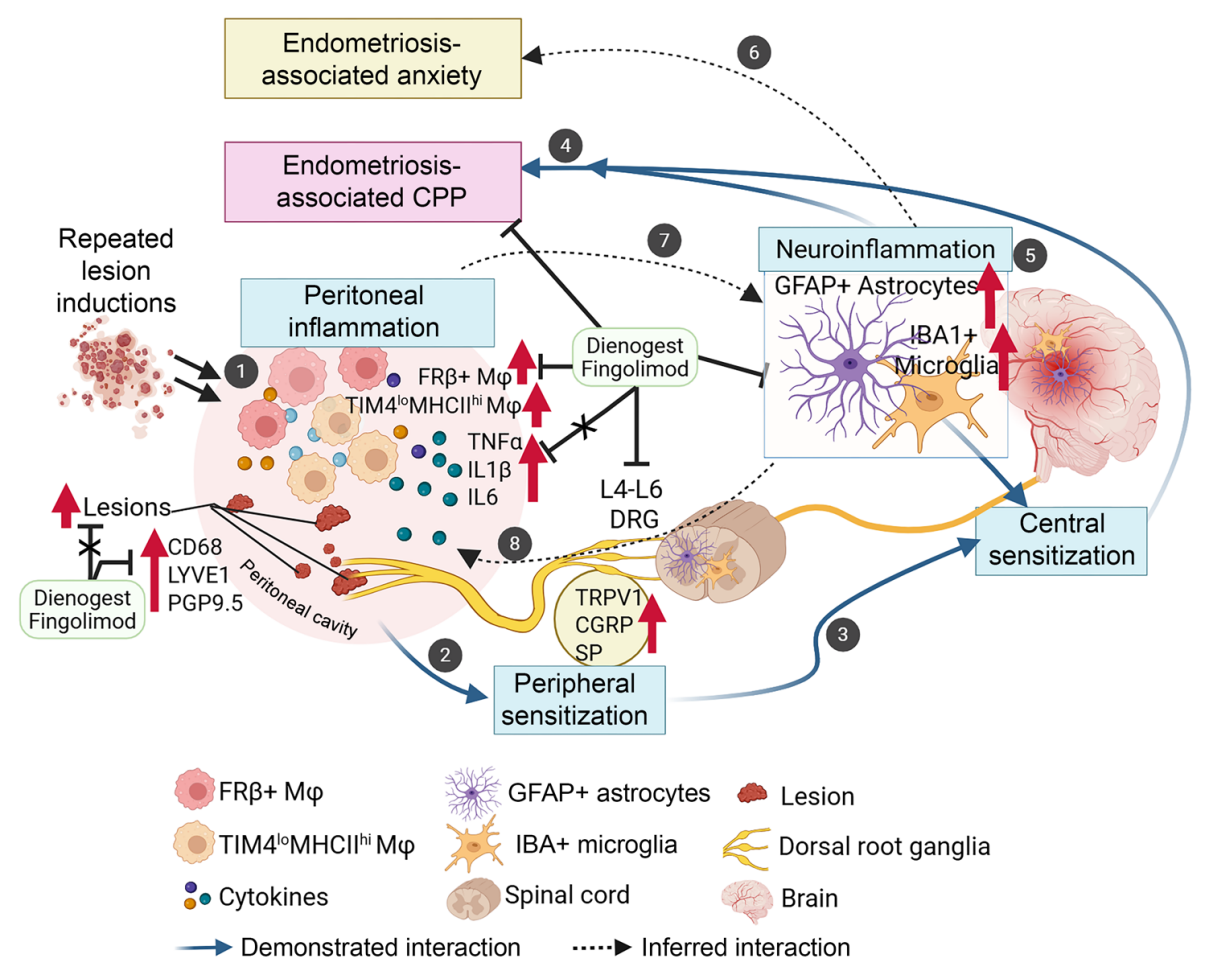
Multiple lesion induction increased chronic inflammation and neuroinflammation, enhancing endometriosis-associated CPP. Created by Biorender. Multiple inductions enhance peripheral sensitization via peripheral chronic inflammation and altered peritoneal macrophage profiles (circle 1). Peripheral inflammation sensitizes nociceptor neurons in endometriotic lesions and/or in pelvic organs, as well as sensory neurons in the DRG (circle 2). The stimuli further sensitize the CNS (circle 3), leading to chronic endometriosis-associated CPP (circle 4). Multiple inductions of lesions induce persistent glial and astrocyte activation as a sign of neuroinflammation across several brain regions (circle 5) linked to pain processing, anxiety, depression, and stress response (circle 6). Dienogest and fingolimod reduce neuroinflammation and hyperalgesia in our multiple-induction endometriosis model, indicating that neuroinflammation drives endometriosis-associated CPP via central sensitization. While chronic systemic or peripheral inflammatory factors and altered immune cells can disrupt the BBB, promoting glial and astrocyte activation, the mechanisms by which peripheral inflammation drives endometriosis-associated neuroinflammation remain to be studied (circle 7). Neuroinflammation can give feedback to stimulate peripheral organs, potentially inducing widespread pain in patients with endometriosis (circle 8). Thus, recurrent retrograde menstruation can be a peripheral stimulus that induces nociceptive pain and triggers a composite chronic inflammatory response, which may be crucial in causing neuroinflammation and further sensitizing the CNS. The circuits of neuroplasticity, driven by chronic inflammation and peripheral organ stimulation via the neuroinflammation feedback loop, may induce widespread endometriosis-associated CPP.

In the present study, we used a multiple-induction mouse model of endometriosis to mimic the impact of recurrent retrograde menstruation. Repeated induction of lesions led to the exacerbation of chronic inflammation and a condition that mimics the patients’ symptoms. Given their elevated hyperalgesia, we used this model to understand endometriosis-associated CPP via central sensitization driven by neuroinflammation (summarized in Figure 14). We demonstrate that multiple inductions can enhance peripheral sensitization via established peripheral chronic inflammation and altered peritoneal macrophage profiles (1 in Figure 14). Peripheral inflammation can sensitize nociceptor neurons in endometriotic lesions and/or in pelvic organs, as well as sensory neurons in the DRG (2 in Figure 14). The stimuli further sensitize the CNS (3 in Figure 14), leading to chronic endometriosis-associated CPP (4 in Figure 14). We have also found that multiple inductions of lesions induce persistent glial and astrocyte activation as a sign of neuroinflammation across several brain regions (5 in Figure 14) linked to pain processing, anxiety, depression, and stress response (6 in Figure 14). The mouse results were supported by the spontaneous endometriosis observed in rhesus macaques. Furthermore, not only dienogest but also fingolimod reduces neuroinflammation and hyperalgesia in our multiple-induction mouse endometriosis model, indicating that neuroinflammation drives endometriosis-associated CPP via central sensitization (Figure 14). While chronic systemic or peripheral inflammatory factors and altered immune cells can disrupt the BBB, promoting glial and astrocyte activation, the mechanisms by which peripheral inflammation drives endometriosis-associated neuroinflammation remain to be studied (7 in Figure 14).

Neuroinflammation can give feedback to stimulate peripheral organs (8 in Figure 14), potentially inducing widespread pain in endometriosis. Indeed, the multiple-induction mice showed greater endometriosis-associated hyperalgesia than the single-induction mice. Especially, hind paw sensitivity was persistent in the multiple-induction mice. Thus, recurrent retrograde menstruation can be a peripheral stimulus that induces nociceptive pain and triggers a composite chronic inflammatory response, which may be crucial in causing neuroinflammation and further sensitizing the CNS. The circuits of neuroplasticity, driven by chronic inflammation and peripheral organ stimulation via the neuroinflammation feedback loop, may induce widespread endometriosis-associated CPP. It is known that the presence of endometriosis lesions does not fully explain endometriosis-associated CPP, and additional mechanisms underlying CNS dysfunction are crucial to understanding it (62, 77, 78, 96, 97). While many studies focus on lesion formation and development in the pathogenesis of endometriosis, it will be necessary to study the underlying mechanisms of the endometriosis-associated CPP to further understand endometriosis pathophysiology.

### Limitations.

(a) A mouse model of endometriosis generates endometriosis lesions and hyperalgesia. The study does not explain why 10%–15% of women develop endometriosis while others do not. (b) We did not include the nonuterine tissue–inoculated group in the present study, as the tissue do not attach and form lesions (98). (c) Central sensitization and neuroinflammation are not always dependent on endometriosis. (d) Although dienogest treatment improved hyperalgesia and neuroinflammation in the present model, the results do not explain why 5%–10% of endometriosis patients do not show pain, and some patients on various hormone therapies develop pain or central sensitization, but some do not. (e) Additional behavioral studies related to anxiety, depression, and stress response will support the findings seen in endometriosis patients. (f) Neural inflammation and neurobiological analyses, such as electrophysiology and calcium imaging, will further help us understand cellular and molecular mechanisms of central sensitization.

## Methods

### Sex as a biological variable.

As this study focused on endometriosis, only female animals were used.

### Study 1: multiple lesion induction model.

Endometriosis-like lesions or sham control (only injected with PBS) were induced in the recipient mice a single time (1x) or 6 times (6x, at 2-week intervals), as shown in Figure 1A. On day –1 (a day before the lesion induction or naive control), day 14, and day 42 (2 and 6 weeks after the last induction of 1x or 6x inductions), a behavioral test was performed, and then mice were euthanized for sample collections: peritoneal fluid was recovered by lavage (4 mL × 2 of ice-cold PBS with 3% FBS), and lesions, bilateral lumbar (L4–6) DRG, spinal cord (L4–6), and brain were collected for further analysis.

### Study 2: multiple lesion induction model with dienogest and fingolimod.

Endometriosis-like lesions were induced in the recipient mice a single time (1x) and 6 times (6x). Mice were randomly assigned to 3 groups and treated daily: (a) PBS vehicle drug control i.p., (b) dienogest (Cayman 21257) 1 mg/kg/b.w. orally by placing a pipette tip containing a dose in PBS into the mouth, and (c) fingolimod (Cayman 10006292) 1 mg/kg/b.w. i.p. for 3 weeks from 3 weeks after the last induction of 1x or 6x inductions, as shown in Figure 10A. A behavior test was performed on day –1 (the day before lesion induction), day 21 (before the treatment), and day 49 (a week after the last treatment to recover from the stress of treatment handling). Mice were then euthanized for sample collection following study 1. Note: PBS was given i.p., as i.p. administration causes greater stress in mice than oral administration.

### Statistics.

Statistical analyses were performed using GraphPad Prism (version 9.5). Data were tested for normal distribution using the Shapiro-Wilk normality test. If data were normally distributed, 1-way ANOVA followed by Tukey’s multiple-comparison test or an unpaired 2-tailed *t* test was used to analyze the differences among the groups or between the 2 groups, respectively. If data were not normally distributed, the Kruskal-Wallis or Mann-Whitney *U* test was performed. The tests used for each figure are stated in the figure legends. A *P* value less than 0.05 was considered statistically significant.

### Study approval.

All mouse experiments were performed at Washington State University (approved by protocol 6751) and according to the NIH *Guide for the Care and Use of Laboratory Animals* (National Academies Press, 2011). Rhesus macaque samples were from the Oregon National Primate Research Center (ONPRC) Macaque Tissue Distribution Program from animals housed at ONPRC under animal assurance protocol #A3304-01.

### Data availability.

Values for all data points in graphs are reported in the Supporting Data Values file. Several methods, including statistical analysis, are described in the Supplemental Methods.

## Author contributions

MS and KH designed the research. MEH, MS, YO, TMP, DAM, and AL performed the experiments or analyzed the data. MS finalized the results and prepared the graphical summary and abstract. JAM, ODS, and AS provided critical feedback on the manuscript. KH wrote the paper; all authors read, reviewed, edited, and approved the manuscript.

## Conflict of interest

The authors have declared that no conflict of interest exists.

## Funding support

This work is the result of NIH funding, in whole or in part, and is subject to the NIH Public Access Policy. Through acceptance of this federal funding, the NIH has been given a right to make the work publicly available in PubMed Central.

- NIH, R01HD104619 to KH.
- NIH, P51OD011092 to ODS.

## Supplementary Material

Supplemental data

Supporting data values

## Version 1 03/17/2026

In-Press Preview

## Version 2 05/15/2026

Electronic publication
