# Supplementary material for: Repeatedly occurring retrograde menstruation intensifies central sensitization driven by neuroinflammation in endometriosis models
Source: J Clin Invest. 2026 Mar 17;136(10):e194136. doi: 10.1172/JCI194136 (PMC13178660; doi:10.1172/JCI194136)
Supplement: Supplemental data [file jci-136-194136-s203.pdf]

Supplementary information

Animals

C57BL/6 mice were purchased from Inotiv and housed in an environment-controlled animal facility (12:12 light-dark cycle) with ad libitum access to food and water. All murine animal experiments were performed at Washington State University in accordance with the NIH guidelines for the care and use of laboratory animals (protocol #6751).

Rhesus macaque samples were obtained from the Oregon National Primate Research Center (ONPRC) Macaque Tissue Distribution Program. Paraffin-embedded blocks of rhesus macaque prefrontal cortex were processed after brain perfusion at necropsy with 4% paraformaldehyded and embedded in paraffin. Eutopic and ectopic endometrial tissues with/without endometriosis were also obtained at the necropsy. The tissue blocks consisted of 12 macaques with advanced chronic endometriosis (some of which presented adenomyosis) and 8 endometriosis-free animals (see details of each animal in Supplementary Table S1). The animals were 5-26 years of age. The macaques with spontaneous endometriosis were first suspiciously identified in the ONPRC research colony by veterinarians in the Department of Comparative Medicine based on symptoms associated with pelvic pain during menses. The animals with clinical signs underwent abdominal palpation and ultrasound. The animals with suspected endometriosis were euthanized and necropsied within 90 days of the assigned project. The animals used in the study were diagnosed with endometriosis based on visual inspection and histopathology (see details of animal diagnosis regimen in Supplementary Information). Endometriosis-free animals were confirmed at necropsy and consisted of female animals in non-endometriosis-associated studies. All animals were pair/group-housed for social enrichment at the ONPRC under animal assurance protocol #A3304-01.

Macaque diagnosis regimen

In the rhesus macaque study, we analyzed samples from ectopic lesions and eutopic endometrium, as well as brain tissues with/without spontaneously developed endometriosis. Endometriosis animals were euthanized based on their advanced condition, which explains the wide range of animal ages. The age range for animals with endometriosis was 7-18 years, and the age range for the controls was 5-26 years. The presence of endometriotic lesions was visually confirmed at necropsy, and lesions were then histologically confirmed by pathologists. The tissue samples listed above were obtained at the same time as the necropsy. Only pathologist-confirmed animals were used in the study as endometriosis-macaques. Endometriosis-free animals were confirmed at necropsy and consisted of female animals in non-endometriosis-associated studies.

Before the euthanasia of endometriosis-macaques, some animals had already shown clinical signs of endometriosis, including heavy or prolonged menses, lethargy, anorexia, weight loss, and abdominal guarding. Rhesus macaques with significant endometriosis can display clinical signs that coincide with the day prior to or the start of menses, including vomiting, anorexia, and scant stool, and these symptoms may improve after menstruation is complete. Narrow-diameter stool, along with clinical symptoms and risk factors, is common in animals with significant endometriosis-related adhesions. Note: We are aware that some animals with active endometriosis may show no clinical signs and have regular menstrual cycles. Those animals were not included in this study as they were not assigned for necropsy.

Animals with the above clinical signs underwent abdominal palpation. Typical signs of endometriosis on palpation may include uterine adhesions, an enlarged or asymmetric uterus, an abnormally firm uterus, or the presence of palpable cystic lesions. Bimanual palpation is the most effective diagnostic modality for identifying uterine changes as early as possible. Then, animals also underwent an ultrasound exam. Ultrasonographic evidence of endometriosis commonly consists of uterine abnormalities in combination with the presence of hypoechoic/cystic structures in the vicinity of the uterus. However, because cysts may be tiny and located anywhere in the body cavity, any abnormal ultrasonographic findings in combination with abnormal uterine palpation should be considered suspect for endometriosis. In addition, animals with endometriosis may not exhibit ultrasonographic changes early in the disease process.

At this point, the animals with suspected endometriosis were assigned to a project associated with the disorder, and they were euthanized and went to necropsy. They were not scheduled for a laparoscopic surgery because this would restrict the animal from being assigned to studies with surgery approval. Animals then underwent a second ultrasound to assess cyst size and the presence of adhesions. After the second ultrasound, the animals scheduled for necropsy were considered for the study. The animals with suspected endometriosis were euthanized and necropsied within 90 days of the assigned project. The animals used in the study were diagnosed with endometriosis based on visual inspection and histopathology.

Mouse model of endometriosis

An experimental mouse model of endometriosis was employed by adopting a published procedure with minor modifications (1). To induce endometriosis-like lesions, female mice (donor) were injected subcutaneously with pregnant mare serum gonadotropin (PMSG, 5 IU, Sigma) to stimulate an estrogenic response within the uterus. Uteri were harvested from donor mice 41 hours after PMSG injection. The endometrium was then separated from the myometrium and dissected into fragments (1-2 mm per side), and 50 mg of fragments were introduced via injection (in 200 μl of PBS) into the peritoneal cavity in the ovary-intact recipient under anesthesia via inhaled isoflurane.

Von Frey test

A standard behavioral (mechanical sensitivity) test was performed before sample collection, as described by our laboratory previously (2, 3). Mice (n=10/group in Study 1, n=6/group in Study 2) were allowed to acclimate in the testing room for 30 min, and then the von Frey test was performed using von Frey filaments (BIO-VF-M, Bioseb). Filaments were applied 10 times to the skin perpendicular to the lower abdomen and bilateral hind paws. The force in grams (g) of the filament evoking a withdrawal response (50% response count as sensitive) was recorded. Three behaviors were considered positive responses to filament stimulation: 1) sharp retraction of the abdomen, 2) immediate licking and/or scratching of the area of filament stimulation, or 3) jumping. All behavioral tests were performed blindly without describing the identity and details of treatment groups to investigators assessing pain. These data were then analyzed by another blinded investigator.

Flow cytometry

Single-cell suspensions of peritoneal exudate cells were used for analyzing immune cell profiles by flow cytometry as described previously (2-5). Briefly, peritoneal exudate cells were lysed using Red Blood Cell Lysis Buffer (BioLegend) and incubated at room temperature for 20 min with Zombie Aqua™ Fixable Viability dye (Bio-Legend). The cells were blocked on ice for 20 min with Fc Block anti-CD16/CD32 (ThermoFisher) and stained with fluorochrome-conjugated monoclonal antibodies for 1 hour (Supplementary Table S2). Samples (n=5/group in Study 1 and n=6/group in Study 2) were acquired with the Attune NxT Acoustic Focusing Cytometer using Attune NxT software (ThermoFisher), and data were analyzed with FlowJo v10.4 software (FLOWJO).

IQELISA

Total protein yield from peritoneal fluid was determined by BCA assay (Pierce), and TNFα (IQM-TNFA-1), IL-1β (IQM-IL1b-1), and IL-6 (IQM-IL6-1) were further quantified by IQELISA kits (Ray Biotech) according to the manufacturer's instructions (n=5/group in Study 1 and n=6/group in Study 2).

Immunohistochemistry

Immunostaining of TRPV1, SP, CGRP, PGP 9.5, LYVE1, IBA1, GFAP, Neurofilament, and CD68 was performed with cross-sections (5 μm) of paraffin-embedded tissues using specific primary antibodies (Supplementary Table S2) and AlexaFluor 488 or 568-conjugated F(ab') secondary antibody (Molecular Probe) or VECTASTAIN ABC kit (Vector lab). Immunostaining images were acquired by Leica DM4 B microscopy. Cell-specific CD68-positive cells were counted and quantified by Image J in the area of 0.289768 mm^2 (n=5/group in Study 1 and n=6/group in Study 2). LYVE1-positive and PGP9.5-positive cells in the lesion were

counted and quantified from three different areas (0.289768 mm^2/area) using Leica LAS X software (n=5/group in Study 1 and n=6/group in Study 2). Neurofilament (NF) was used as a pan-neuronal marker and was co-stained with TRPV1, SP, or CGRP. TRPV1, SP, or CGRP positive DRG neurons in the section were counted in the area of 0.289768 mm^2, and the percentages of TRPV1, SP, or CGRP positive cells per neurofilament-positive DRG were shown (n=5/group in Study 1 and n=6/group in Study 2).

Image analysis

Image analysis for IBA1 and GFAP was performed as described previously (6) with some modifications. Immunostained IBA1 or GFAP images (1.159063 mm^2 in size) of the spinal cord (dorsal horn) and the brain (cortex, hippocampus, thalamus, and hypothalamus) were taken and exported by a blinded researcher to avoid any experimental bias. The exported images (1280x960 pixels) were deconvoluted using the inbuilt "Color Deconvolution (H-DAB)" function in Fiji image analysis software to obtain brown-stained areas (7). The images were loaded into the machine learning "Trainable Weka Segmentation" plugin in Fiji, and the plugin was trained to identify three classes of immunostaining: stained cells, non-stained cells, and background. Then, the images were processed to create a classified image and thresholded (8). The size and the number of cells were measured using the "Analyze Particles" function in Fiji with a size threshold of 45-infinity. The number of cells was divided by the analyzed area. For determining the percentage area, the total area of immunoreactivity was divided by the analyzed area (Mice: n=5/group in Study 1 and n=6/group in Study 2, Macaques: n=3 control and n=8 endometriosis).

Quantitative analysis for neurofilament (NF) in the ectopic lesions (EcE) and eutopic endometrium with (EuE) or without (control) spontaneous endometriosis in rhesus macaques (n=5/group) was performed with Image J. Fluorescent-stained images (1.159063 mm^2 in size) were used and assessed by a blinded researcher. The NF+ GFP (green) layer was extracted by the "Split Channels" function and adjusted with an identical threshold across images. The percentage of NF+ area was then calculated by the "Measure" function under "Analyze".

1. Nothnick WB, Colvin A, Cheng KF, and Al-Abed Y. Inhibition of macrophage migration inhibitory factor reduces endometriotic implant size in mice with experimentally induced disease. *J Endometr.* 2011;3(3):135-42.
2. Herup-Wheeler T, Shi M, Harvey ME, Talwar C, Kommagani R, MacLean II JA, et al. High-fat diets promote peritoneal inflammation and augment endometriosis-associated abdominal hyperalgesia. *Front Endocrinol (Lausanne).* 2024.
3. Shi M, MacLean JA, 2nd, and Hayashi K. The involvement of peritoneal GATA6(+) macrophages in the pathogenesis of endometriosis. *Frontiers in immunology.* 2024;15:1396000.
4. Shi M, Sekulovski N, Whorton AE, MacLean JA, 2nd, Greaves E, and Hayashi K. Efficacy of niclosamide on the intra-abdominal inflammatory environment in endometriosis. *FASEB J.* 2021;35(5):e21584.
5. Zhao L, Shi M, Winuthayanon S, MacLean JA, 2nd, and Hayashi K. Niclosamide targets the dynamic progression of macrophages for the resolution of endometriosis in a mouse model. *Commun Biol.* 2022;5(1):1225.
6. Bashir ST, Redden CR, Raj K, Arcanjo RB, Stasiak S, Li Q, et al. Endometriosis leads to central nervous system-wide glial activation in a mouse model of endometriosis. *J Neuroinflammation.* 2023;20(1):59.
7. Schindelin J, Arganda-Carreras I, Frise E, Kaynig V, Longair M, Pietzsch T, et al. Fiji: an open-source platform for biological-image analysis. *Nat Methods.* 2012;9(7):676-82.
8. Arganda-Carreras I, Kaynig V, Rueden C, Eliceiri KW, Schindelin J, Cardona A, et al. Trainable Weka Segmentation: a machine learning tool for microscopy pixel classification. *Bioinformatics.* 2017;33(15):2424-6.

Supplementary Table S1: Summary of rhesus macaque history with/without endometriosis.

Macaques without endometriosis (controls) were used for the analysis of eutopic endometrium.

Subject #	Reproductive History[1] # live births	Onset of clinical symptoms In days before sample collection	Age at sample collection (years; days)	Menstrual cycle history in days (mean $\pm$ SD)[2]	Cycle day (phase) @ collection	Pathology notes:
Rh034	None	n/a	11y; 330d	31.33 $\pm$10.54	Day 23 (secretory)	Endometriosis-free and no clinical signs of endometriosis. The animal displayed an oviductal cyst with clear fluid and tubal occlusion.
Rh044	7 births @ 6y; 4d 7y; 57d 8y; 71d 9y; 43d 11y,11d 12y,87d 14y,12d	n/a	26y; 10d	44.3111 $\pm$ 24.87	Day 26 (secretory)	Endometriosis-free and no clinical signs of endometriosis.
Rh168	None	n/a	6y; 11d	35.11 $\pm$ 10.75	Day 41 (secretory)	Endometriosis-free and no clinical signs of endometriosis.
Rh792	None	n/a	5y; 47d	27.77 $\pm$ 3.38	Day 24 (secretory)	Endometriosis-free and no clinical signs of endometriosis.
Rh986	4 births @ 4y; 52d 5y; 68d 7y; 54d 8y; 22d	n/a	18y; 51d	29.67 $\pm$ 6.55	Day 19 (secretory)	Endometriosis-free and no clinical signs of endometriosis.

[1]Reproductive history shows # of live births and the age of the dam in years and days at parturition.

[2]Cycle length was recorded in the breeding season prior to surgery (9-10 cycles per animal).

Macaques with endometriosis were used for the analysis of ectopic and eutopic endometrium.

Subject #	Reproductive History[1] # live births	Onset of clinical symptoms In days before sample collection	Age at sample collection (years; days)	Menstrual cycle history in days (mean $\pm$ SD)[2]	Cycle day (phase) @ collection	Pathology notes:
Rh019	none	214d	7y; 308d	24.88 $\pm$ 3.95	Day 24 (secretory)	Two foci of endometriosis associated with omental fat; and a lesion >5 cm across that completely obliterated the uterine cul-de-sac. No mention of adenomyosis in the record.
Rh444	4 births @ 7y; 2d 8y; 72d 12y; 12d 14y; 145d	176d	15y; 301d	31.34 $\pm$ 8.10	Day 27 (secretory)	Five endometriotic cysts 0.8; 0.5; 0.4; 0.9 and 0.3 cm across were collected between the uterus and colon and the uterus and bladder. Uterus enlarged, irregular, and adenomyosis indicated, confirmed by histology.
Rh491	1 births @ 8y; 68d	146d	8y; 267d	27.12 $\pm$ 2.20	Day 21 (secretory)	Reproductive tract: the omentum was multifocally adhered to the serosal surface of the uterine fundus which was expanded by an ~1.0x1.5 cm dark purple to brown cyst that is encased in abundant adipose tissue and variably sized bands of firm white fibrous connective tissue that entraps bilateral ovaries and encircles the descending colon. No mention of adenomyosis in the record.
Rh503	1 births @ 6y; 38d	147d	7y; 188d	34.11 $\pm$ 7.51	Day 27 (secretory)	Endometriosis with severe adhesions were noted with the ovaries, oviducts, uterus, colon, broad ligament, caudal ureters, and the urinary bladder were enmeshed and partially to completely obscured by an endometriotic cyst and fibrous connective tissue. A bleeding endometriotic cyst >5 cm surrounding the uterus, oviducts and ovaries was removed en bloc for the investigator. No mention of adenomyosis in the record.
Rh818	1 births @ 6y; 322d	81d	8y; 142d	30.26 $\pm$ 3.68	Day 10 (proliferative)	A 3-6 cm diameter dark brown fluid-filled cystic structure on the dorsal aspect of the uterus was removed en bloc for the investigator. The endometriotic cyst involved the uterus, ovaries, and oviducts with no evidence of perforation. Uterus enlarged, irregular, and adenomyosis indicated, confirmed by histology.

[1]Reproductive history shows # of live births and the age of the dam in years and days at parturition.

[2]Cycle length was recorded in the breeding season prior to surgery (9-10 cycles per animal).

Macaques without endometriosis (controls) were used for brain analysis.

Subject #	Reproductive History[1] # live births	Onset of clinical symptoms In days before sample collection	Age at sample collection (years; days)	Menstrual cycle history in days (mean $\pm$ SD)[2]	Cycle day (phase) @ collection	Pathology notes:
Rh025	None	n/a	9y,306	30.10 $\pm$ 6.10	Day 12 (proliferative)	Endometriosis-free and no clinical signs of endometriosis. Chronic weight loss; diarrhea.
Rh708	None	n/a	13y,28d	28.13 $\pm$ 1.20	Day 3 (menstruating)	Endometriosis-free and no clinical signs of endometriosis. Chronic weight loss; diarrhea; diagnosed heart murmur
Rh880	None	n/a	7y,168d	27.03 $\pm$ 3.6	Day 3 (menstruating)	Endometriosis-free and no clinical signs of endometriosis. Chronic diarrhea.

[1]Reproductive history shows # of live births and the age of the dam in years and days at parturition.

[2]Cycle length was recorded in the breeding season prior to surgery (9-10 cycles per animal).

Macaques with endometriosis were used for brain analysis.

Subject #	Reproductive History[1] # live births	Onset of clinical symptoms In days before sample collection	Age at sample collection (years; days)	Menstrual cycle history in days (mean ± SD)[2]	Cycle day (phase) @ collection	Pathology notes:
Rh153	4 births @<br>4y; 102d<br>5y; 106d<br>6y; 49d<br>8y; 41d	203d	17y; 299d	30.11 ± 1.65	Day 28 (secretory)	A >3 cm endometriotic mass bridging the colon and uterus and 6 small lesions <0.3cm. Adhesions of omentum and left ovary and oviduct to the uterine fundus were observed in addition to the lesions. No mention of adenomyosis in the record.
Rh155	4 births @<br>4y; 39d<br>5y; 10d<br>6y; 52d<br>8 y; 78d	289d	17y; 8d	26.33 ± 0.07	Day 24 (secretory)	Omentum on the right side of the abdomen contained a 7 cm x 6 cm x 3 cm firm fibrotic/ endometriotic mass with multiple cystic spaces containing thick red-brown fluid. At collection, 6 additional endometriotic cysts noted; 3 omental, 2 on the peritoneum, and one on the uterine wall. Uterus irregular with an echogenic mass consistent with adenomyosis, but not confirmed histologically.
Rh271	3 births @<br>4y; 71d<br>6y; 72d<br>8y; 44d	294d	17y; 10d	29.33 ± 1.76	Day 30 (pre-menstrual)	Reproductive tracts: ovaries, oviducts, uterus, cervix, and vagina were collected en bloc due to a large >6 cm ruptured endometriotic cyst. Adenomyosis confirmed by uterine ultrasound and histology.
Rh366	1 birth @<br>5y; 91d	232d	13y; 290d	25.55 ± 1.87	Day 23 (secretory)	A large >3 cm ruptured endometriotic cyst was observed surrounding the uterus, ovaries, and oviducts. This massed tissue was removed en bloc for the investigator. No mention of adenomyosis in the record.
Rh367	5 births @<br>4y; 60d<br>6y; 4d<br>7y; 55d<br>8y; 4d<br>9y; 46d	114d	18y; 272 d	27.11 ± 3.76	Day 24 (secretory)	The reproductive tract was resected en bloc due to endometriosis. Multiple endometriosis lesions in the omentum and on the uterus and oviducts; 10 lesions <0.5 cm in diameter were collected. Abnormal uterus noted, adenomyosis suspected but not confirmed histologically.
Rh490	none	151d	13y; 167d	35.71 ± 7.68	Day 46 (secretory)	Multiple endometriosis lesions, the largest of which measures approximately 1.5 x 1.5 x 1.5 cm were identified in the caudal peritoneal cavity; and adhered to the dorsal bladder wall and uterus. Additional endometriosis foci and multiple adhesions between viscera, mesentery, omentum, and the body wall. Uterine adenomysosis evident, confirmed histologically.
Rh543	3 births @<br>4y; 8 d<br>5y; 44d<br>6 y; 73d	403d	17y; 167d	25.44 ± 1.87	Day 3, (menstruating)	At collection, a 3 cm endometriotic cyst between the colon and uterus, plus one on the bladder; clear evidence of adneomyosis in the myometrium on the ventral wall of the uterus, but not confirmed histologically.

[1]Reproductive history shows # of live births and the age of the dam in years and days at parturition.

[2]Cycle length was recorded in the breeding season prior to surgery (9-10 cycles per animal).

Supplementary Table S2 Antibodies and Reagents for Flow Cytometry and Immunochemistry

Antibody	Conjugate	Company	Catalog#	Application
CD11b (M1/70)	APC-Cy7	BD Biosciences	557657	Flow
CD19 (ID3)	NovaFluor Yellow 610	eBioscience™	M004T02Y03	Flow
CD3 (17A2)	FITC	BioLegend	100203	Flow
CD45 (30-f11)	PE-Cy5	BioLegend	103109	Flow
CD68 (KP1)	Unconjugated	Abcam	ab955	IHC
CGRP (4901)	Unconjugated	Abcam	ab81887	IHC
GFAP	Unconjugated	Abcam	ab7260	IHC
IA/IE, MHC II (M5/114)	BV711	BD Biosciences	563414	Flow
IBA1	Unconjugated	Wako	019-19741	IHC
Ly6G (1A8)	Super Bright 436	eBioscience™	62-9668-82	Flow
LYVE1	Unconjugated	AngioBio	11-034	IHC
Neurofilament	Unconjugated	Millipore	AB5539	IHC
PGP9.5	Unconjugated	Invitrogen	PA5-29012	IHC
Substance P (SP-DE4-21)	Unconjugated	Abcam	ab14184	IHC
TIM4 (RMT4-54)	PE	BioLegend	130005	Flow
TRPV1 (VR1-C)	Unconjugated	Neuromics	RA14113	IHC
Fc Block CD16/CD32 antibody		Thermo Fisher	14-0161-82	Flow
Total Antibody Compensation Bead Kit		Thermo Fisher	A10513	Flow
Zombie Aqua™ Fixable Viability Kit		BioLegend	423101	Flow

Supplementary Figures

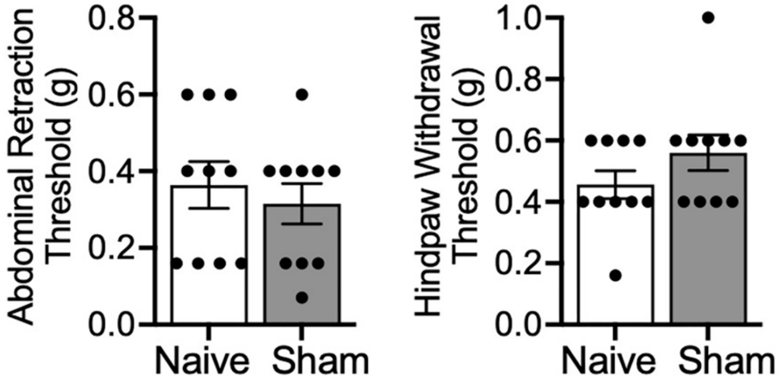

Supplementary Figure S1. **Evaluation of endometriosis-associated hyperalgesia between naïve (Day -1: a day before the lesion induction) and sham (multiple PBS injections at 6 weeks).** Abdominal and hind paw withdrawal thresholds were assessed using the von Frey test. Data were analyzed using the Mann-Whitney test, following the Shapiro-Wilk normality test. Data are shown as mean ± SEM (n = 10). No significant difference was detected.

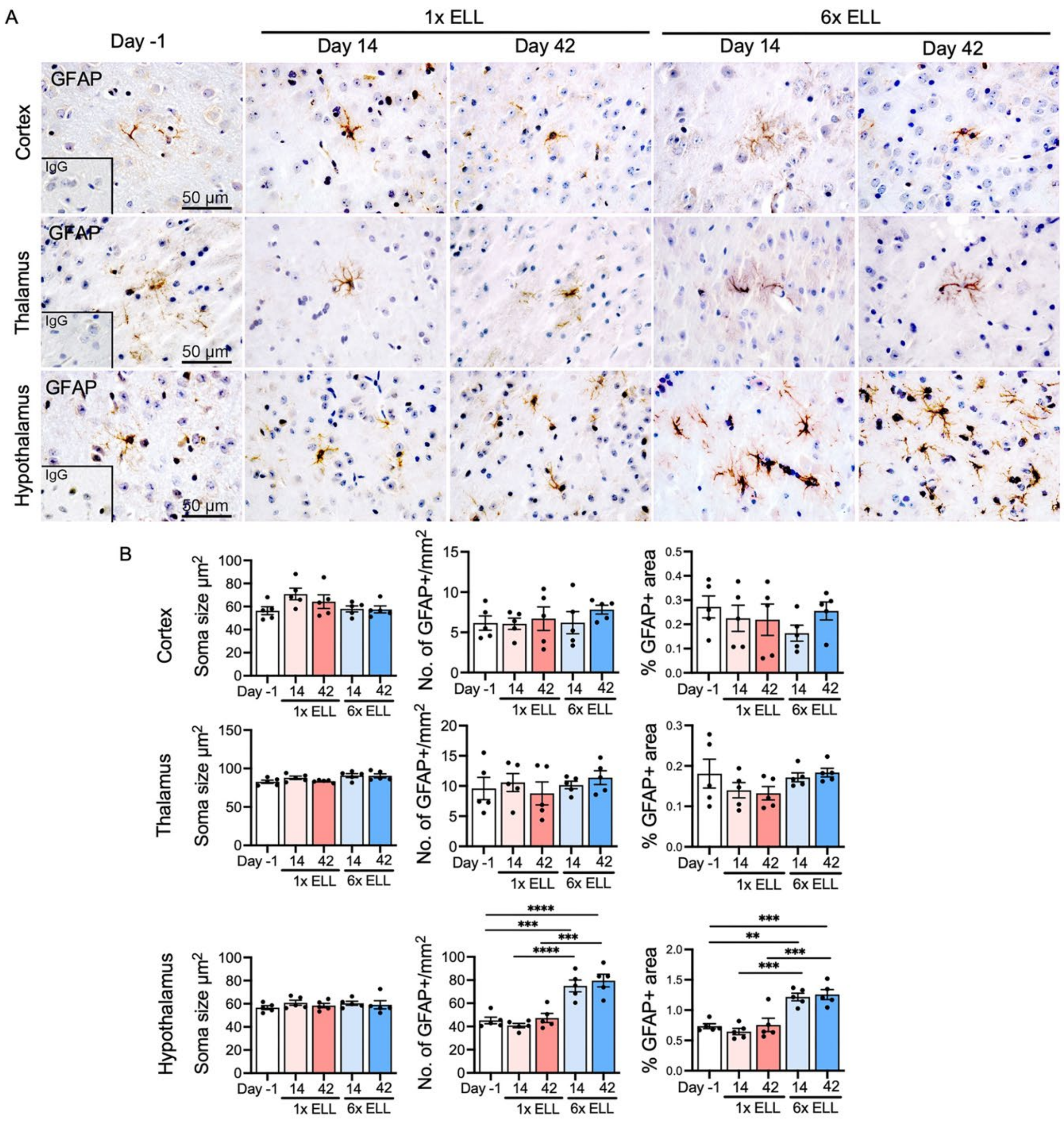

Supplementary Figure S2. **Representative immunohistochemical images (A) and quantification (B) of GFAP in the cortex, thalamus, and hypothalamus in the single- or multiple-induction mice at 2 or 6 weeks after the last lesion induction.** Following the Shapiro-Wilk normality test, one-way ANOVA followed by Tukey's multiple-comparison test was used to analyze differences among groups. Data are shown as the mean ± SEM (n=5). ELL: endometriosis-like lesions. ***P* < 0.01, ****P* < 0.001, *****P* < 0.0001.

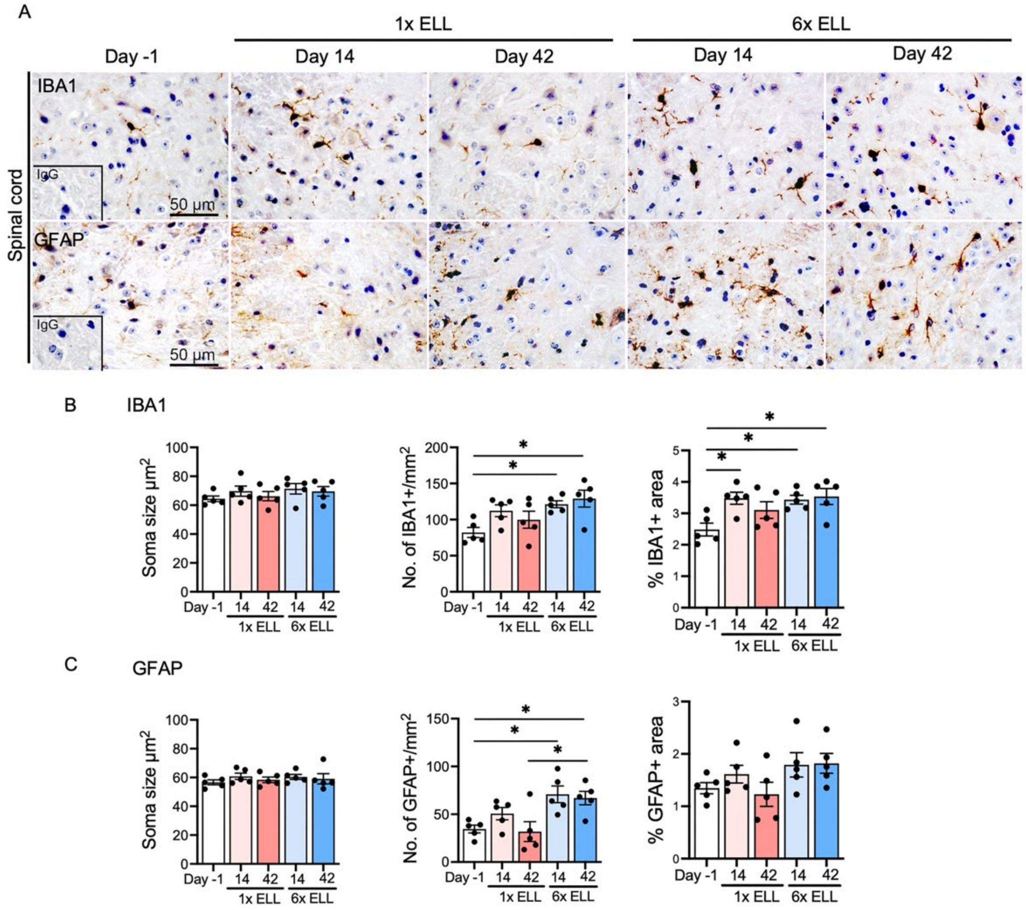

Supplementary Figure S3. **Representative immunohistochemical images (A) and quantification (BC) of IBA1 and GFAP in the spinal cord in the single- or multiple-induction mice at 2 or 6 weeks after the last lesion induction.** Following the Shapiro-Wilk normality test, one-way ANOVA followed by Tukey's multiple-comparison test was used to analyze differences among groups. Data are shown as the mean ± SEM (n=5). ELL: endometriosis-like lesions. *P < 0.05.

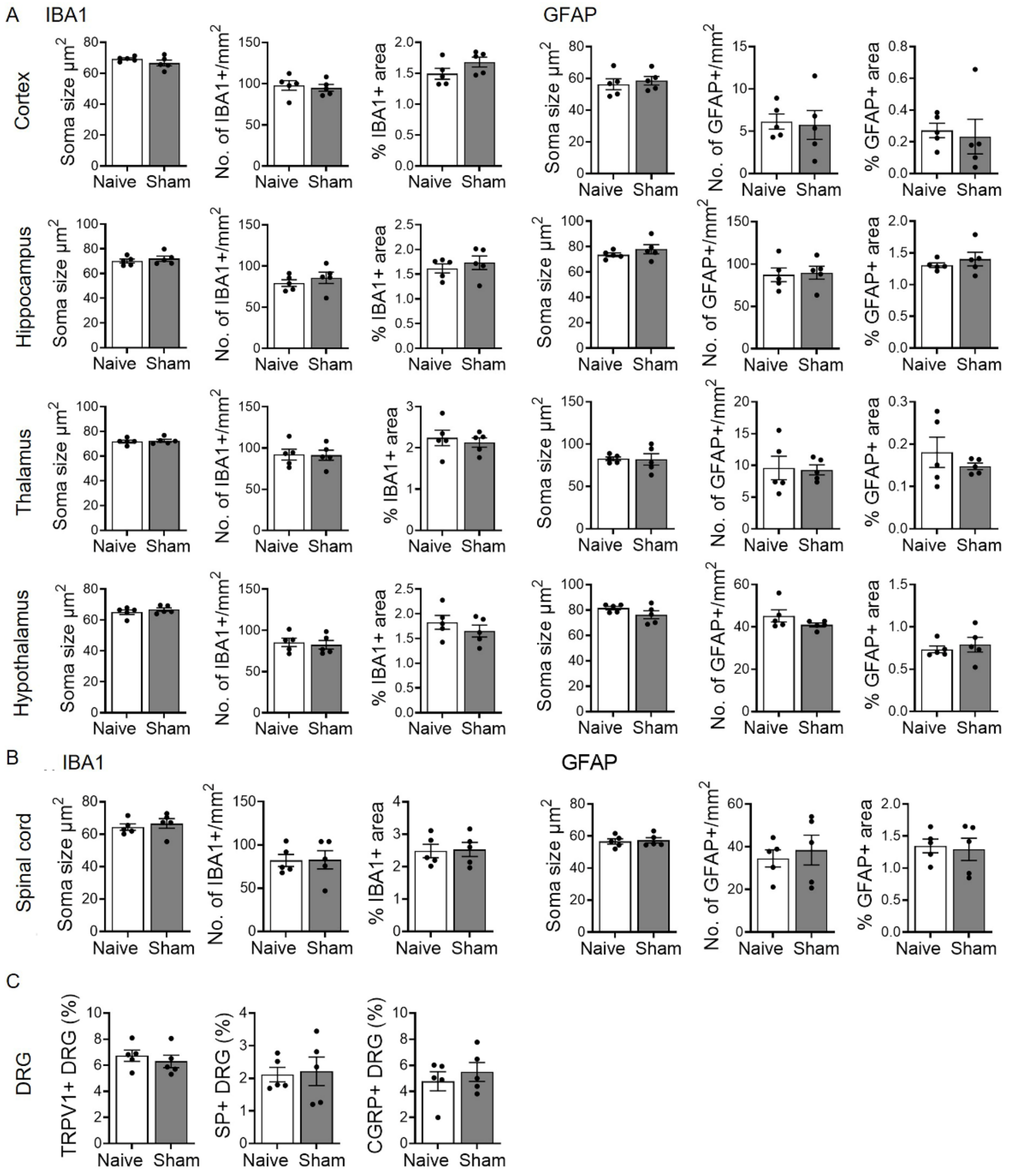

Supplementary Figure S4. **Evaluation of IBA1 and GFAP in the cortex, hippocampus, thalamus, hypothalamus (A), spinal cord (B), and DRG (C) between naïve (Day -1: a day before the lesion induction) and sham (multiple PBS injections at 6 weeks).** Following the Shapiro-Wilk normality test, an unpaired two-tailed t-test was used to analyze group differences. Data are shown as the mean ± SEM (n=5). No significant difference was detected.

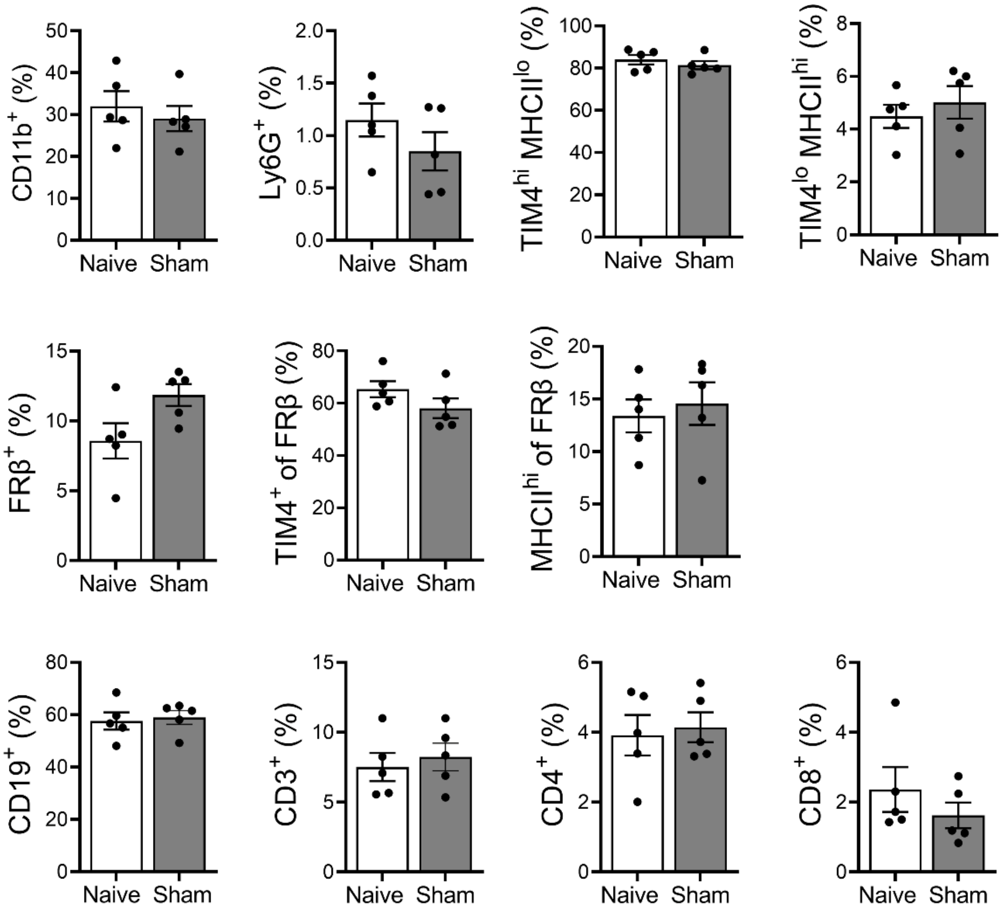

Supplementary Figure S5. **Evaluation of immune cell profiles in the peritoneal cavity between naïve (Day -1: a day before the lesion induction) and sham (multiple PBS injections at 6 weeks).** Following the Shapiro-Wilk normality test, an unpaired t-test was used to analyze group differences. Data are shown as the mean ± SEM (n=5). No significant difference was detected.

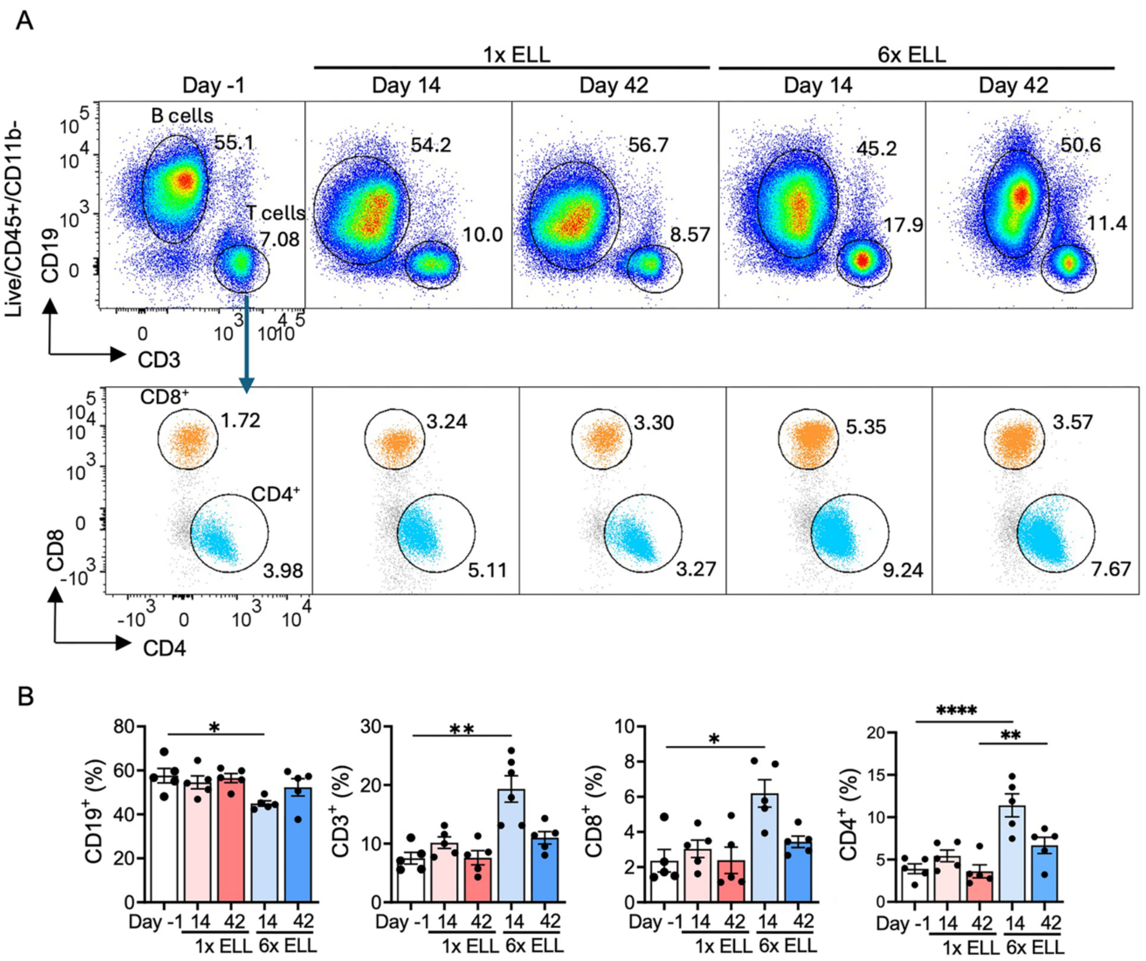

Supplementary Figure S6. **Comparison of peritoneal B or T cell profiles in single- or multiple-induction mice at 2 or 6 weeks after the last lesion induction.** (**A**) Representative flow plots illustrating the composition of CD19+ and CD3+ cells. CD3+ cells were further gated by CD8 and CD4. (**B**) Proportions of CD19+ or CD3+ and CD8+ or CD4+ are shown. Following the Shapiro-Wilk normality test, one-way ANOVA followed by Tukey's multiple-comparison test was used to analyze CD19+ cells among groups, whereas the Kruskal-Wallis test was used to assess group differences in CD3+, CD8+, and CD4+ cells. Data are shown as the mean ± SEM (n=5). ELL: endometriosis-like lesions. *$P < 0.05$, **$P < 0.01$, ****$P < 0.0001$.

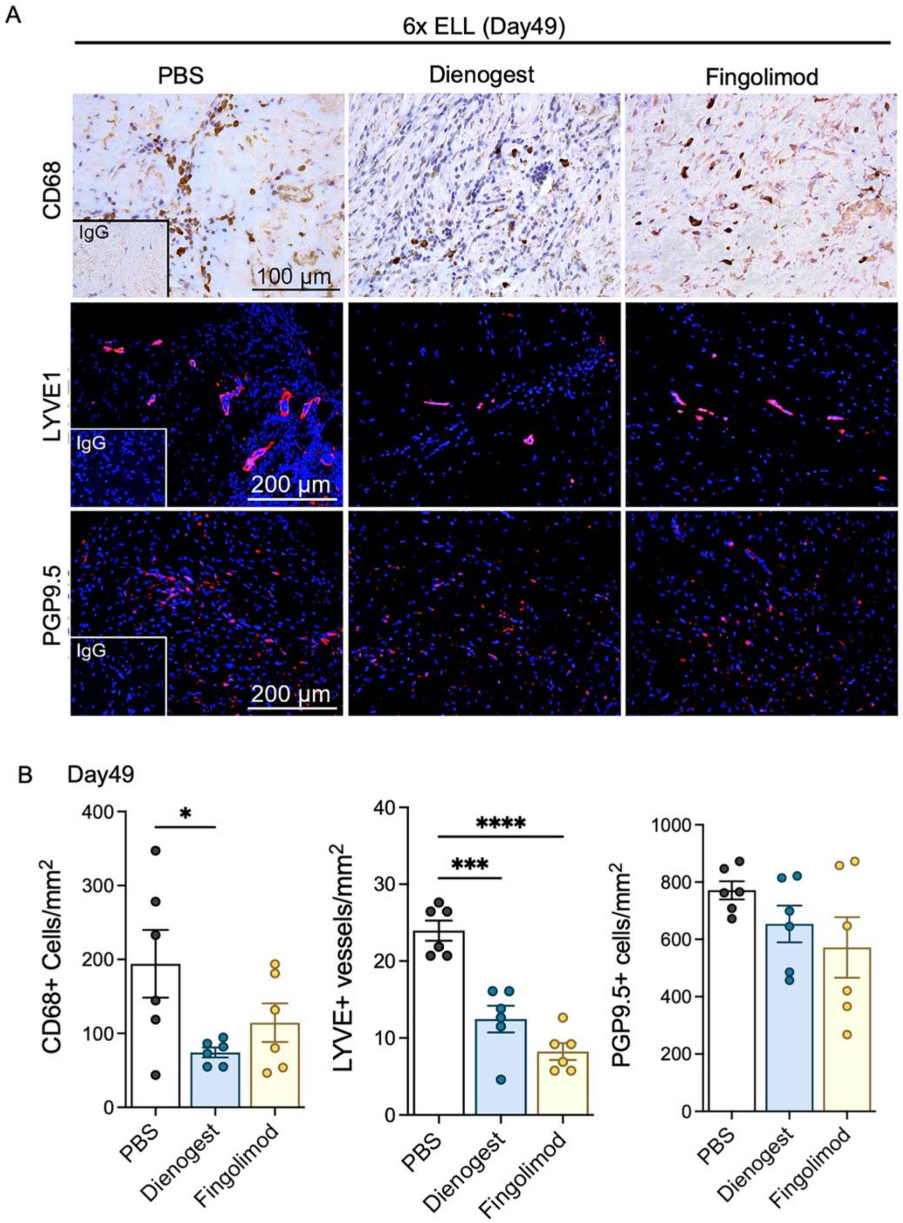

Supplementary Figure S7. **Representative immunohistochemical images (A) and quantification (B) of CD68+, LYVE1+, or PGP9.5+ cells in the lesions (n=6) following dienogest or fingolimod treatment in the multiple-induction mice.** Following the Shapiro-Wilk normality test, one-way ANOVA followed by Tukey's multiple-comparison test was used to analyze the numbers of CD68+, LYVE1+, or PGP9.5+ cells in the lesions among groups. Data are shown as the mean ± SEM (n=6). ELL: endometriosis-like lesions. *$P < 0.05$, ***$P < 0.001$,

****$P < 0.0001$.

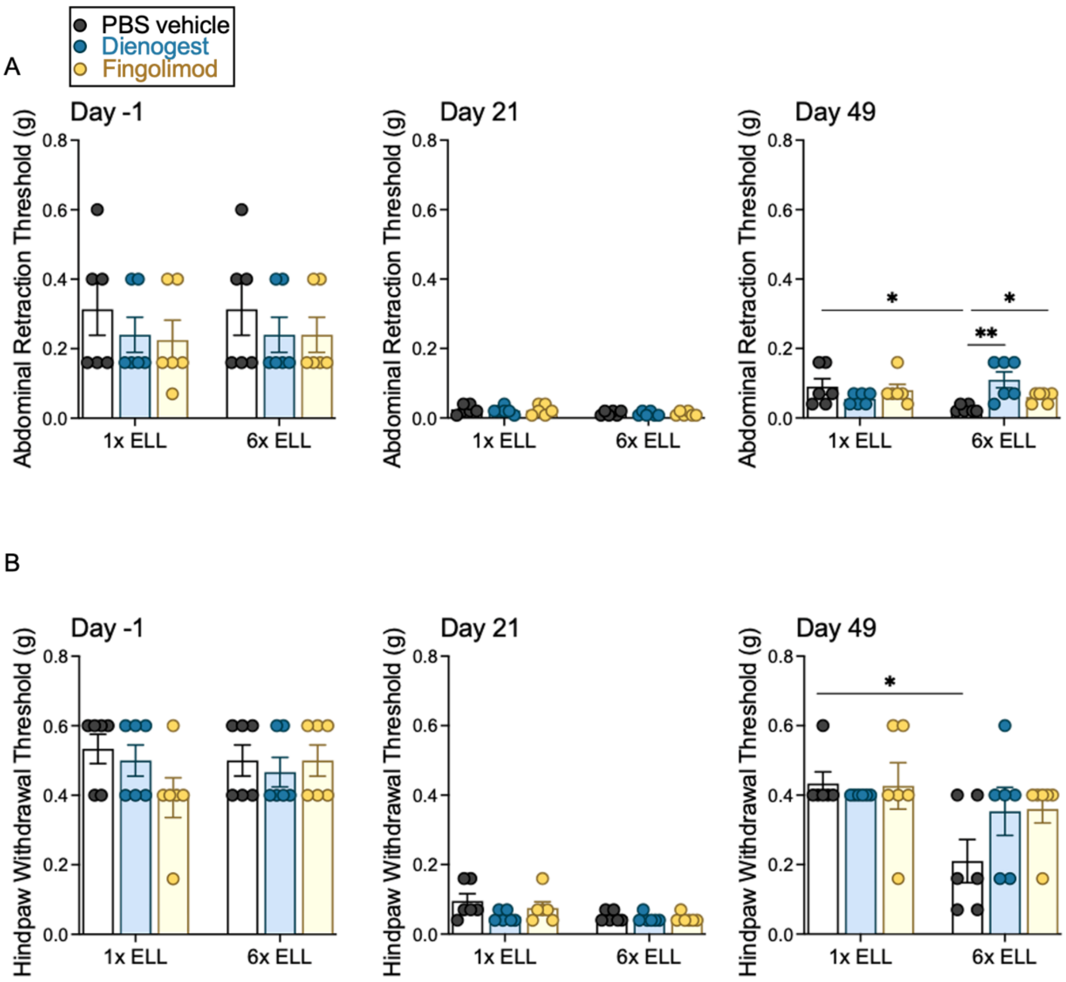

Supplementary Figure S8. **Comparison of the abdominal (A) and hind paw withdrawal (B) thresholds between single and multiple inductions, as well as the dienogest and fingolimod treatment groups at each time point (n=6).** Following the Shapiro-Wilk normality test, differences between single and multiple induction within the single group were compared using the Mann-Whitney test, whereas differences between treatment groups were compared with the Kruskal-Wallis test. Data are shown as the mean ± SEM. ELL: endometriosis-like lesions. *$P < 0.05$, **$P < 0.01$.

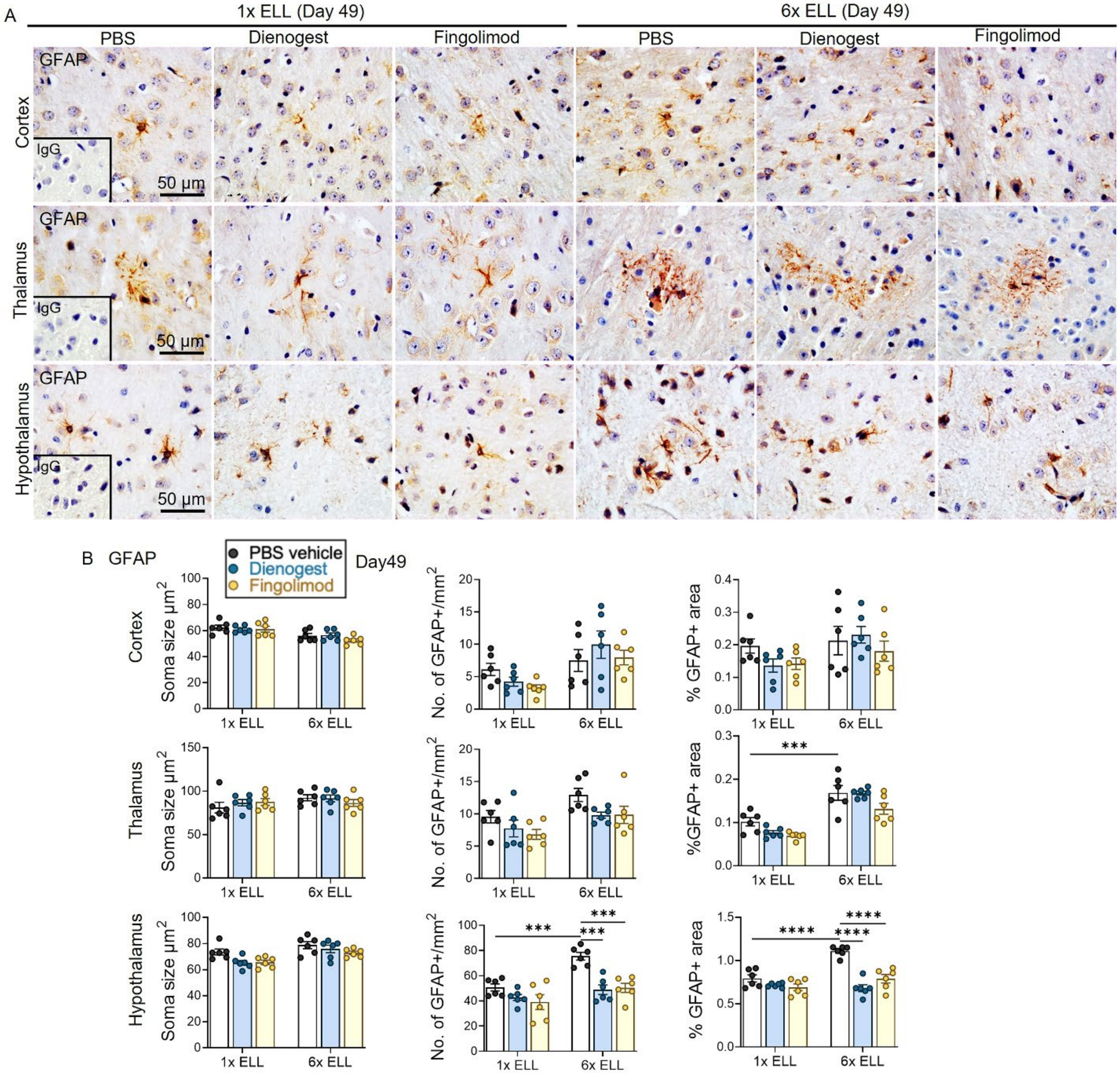

Supplementary Figure S9. **Representative immunohistochemical images (A) and quantification (B) of GFAP in the cortex, thalamus, and hypothalamus in the single- or multiple-induction mice followed by dienogest or fingolimod treatment.** Following the Shapiro-Wilk normality test, one-way ANOVA followed by Tukey's multiple-comparison test was used to analyze differences among groups. Data are shown as the mean ± SEM (n=6). ELL: endometriosis-like lesions. ***$P < 0.001$, ****$P < 0.0001$.

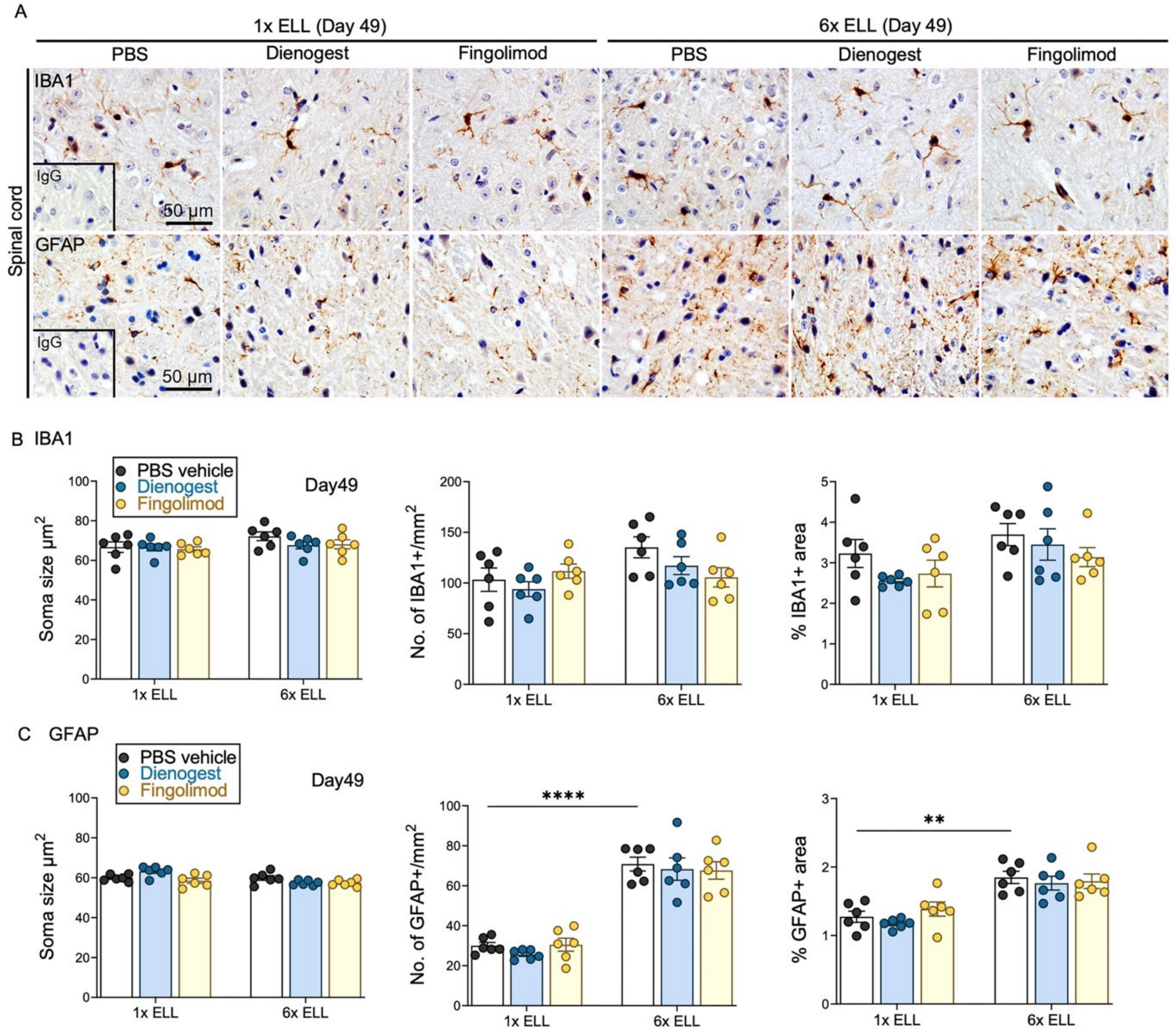

Supplementary Figure S10. Representative immunohistochemical images (A) and quantification (BC) of IBA1 and GFAP in the spinal cord in the single- or multiple-induction mice followed by dienogest or fingolimod treatment. Following the Shapiro-Wilk normality test, one-way ANOVA followed by Tukey's multiple-comparison test was used to analyze differences among groups. Data are shown as the mean ± SEM (n=6). ELL: endometriosis-like lesions. ***P* < 0.01, *****P* < 0.0001.

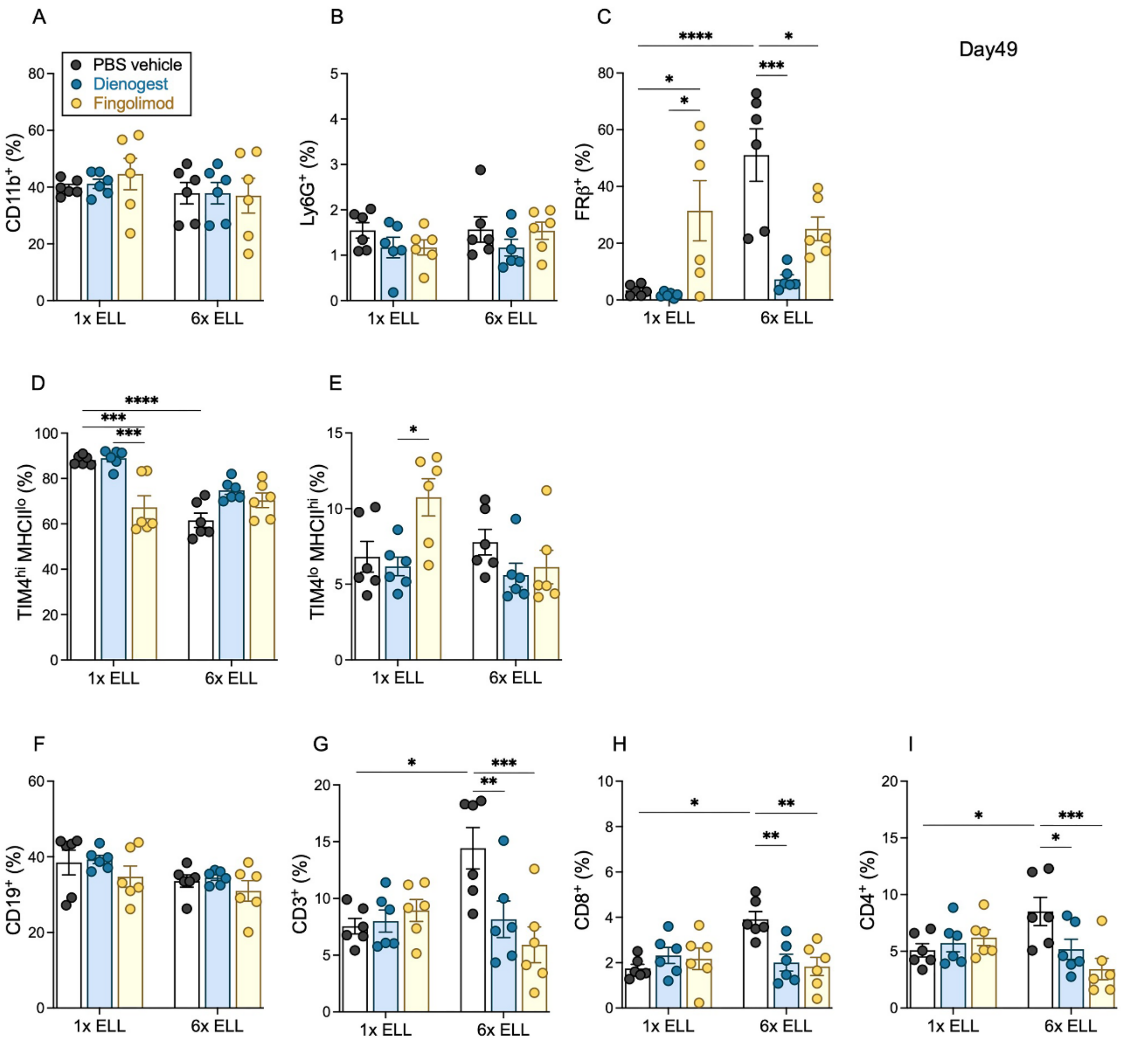

Supplementary Figure S11. The effects of dienogest and fingolimod on the peritoneal immune cell profiles in single- or multiple-induction mice at 7 weeks (Day 49) after the last lesion induction. The same gating strategy was used, as shown in Figure 8. Proportions of CD11b+ cells (**A**), Ly6G+ cells (**B**), FRβ+ of CD11b+ cells (**C**), TIM4^{hi} MHCII^{lo} (**D**) and TIM4^{lo} MHCII^{hi} (**E**) of CD11b+ cells, and CD19+ cells (**F**), CD3+ cells (**G**), CD8+ cells (**H**), and CD4+ cells (**I**) are shown. Following the Shapiro-Wilk normality test, all comparisons among groups were performed using one-way ANOVA followed by Tukey's multiple-comparison test. Data are shown as the mean ± SEM (n=6). ELL: endometriosis-like lesions. *$P < 0.05$, **$P < 0.01$, ***$P < 0.001$, ****$P < 0.0001$.

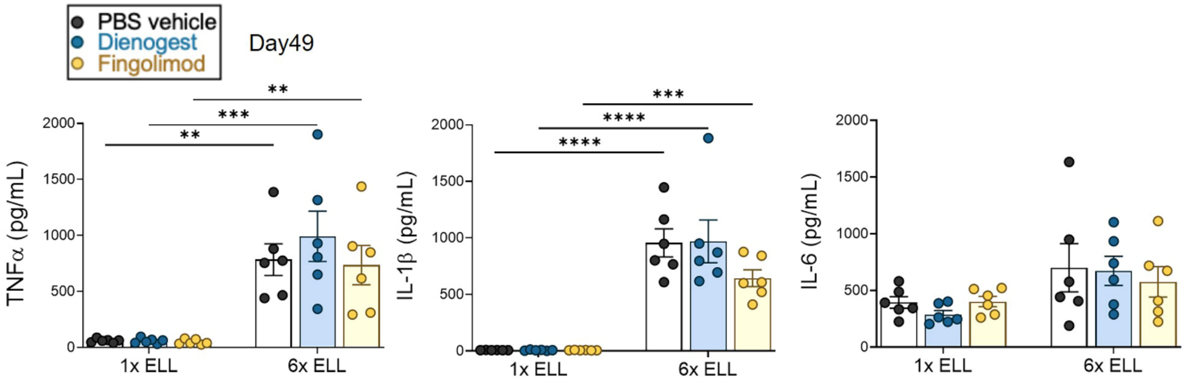

Supplementary Figure S12. The effects of dienogest and fingolimod on the peritoneal cytokine levels (TNFα, IL-1β, and IL-6) in single- or multiple-induction mice at 7 weeks (Day 49) after the last lesion induction. Cytokine levels were analyzed by IQELISA. Following the Shapiro-Wilk normality test, one-way ANOVA followed by Tukey's multiple-comparison test was used to analyze differences among groups. Data are shown as the mean ± SupplementarySEM (n=6). ELL: endometriosis-like lesions. $^{**}P < 0.01$, $^{***}P < 0.001$, $^{****}P < 0.0001$.
